# MicroRNA-mRNA Co-sequencing Identifies Transcriptional and Post-transcriptional Regulatory Networks Underlying Muscle Wasting in Cancer Cachexia

**DOI:** 10.3389/fgene.2020.00541

**Published:** 2020-05-29

**Authors:** Geysson Javier Fernandez, Juarez Henrique Ferreira, Ivan José Vechetti, Leonardo Nazario de Moraes, Sarah Santiloni Cury, Paula Paccielli Freire, Jayson Gutiérrez, Renato Ferretti, Maeli Dal-Pai-Silva, Silvia Regina Rogatto, Robson Francisco Carvalho

**Affiliations:** ^1^Department of Structural and Functional Biology, Institute of Biosciences, São Paulo State University, Botucatu, Brazil; ^2^Faculty of Medicine, University of Antioquia, Medellín, Colombia; ^3^Department of Plant Biotechnology and Bioinformatics, Ghent University, Ghent, Belgium; ^4^Department of Clinical Genetics, University Hospital of Southern Denmark, Vejle, Institute of Regional Health Research, University of Southern Denmark, Odense, Denmark

**Keywords:** Lewis Lung Cancer, miRNAs, transcription factors, extracellular matrix, cancer cachexia

## Abstract

Cancer cachexia is a metabolic syndrome with alterations in gene regulatory networks that consequently lead to skeletal muscle wasting. Integrating microRNAs-mRNAs omics profiles offers an opportunity to understand transcriptional and post-transcriptional regulatory networks underlying muscle wasting. Here, we used RNA sequencing to simultaneously integrate and explore microRNAs and mRNAs expression profiles in the tibialis anterior (TA) muscles of the Lewis Lung Carcinoma (LLC) model of cancer cachexia. We found 1,008 mRNAs and 18 microRNAs differentially expressed in cachectic mice compared with controls. Although our transcriptomic analysis demonstrated a high heterogeneity in mRNA profiles of cachectic mice, we identified a reduced number of differentially expressed genes that were uniformly regulated within cachectic muscles. This set of uniformly regulated genes is associated with the extracellular matrix (ECM), proteolysis, and inflammatory response. We also used transcriptomic data to perform enrichment analysis of transcriptional factor binding sites in promoter sequences, which revealed activation of the atrophy-related transcription factors NF-κB, Stat3, AP-1, and FoxO. Furthermore, the integration of mRNA and microRNA expression profiles identified post-transcriptional regulation by microRNAs of genes involved in ECM organization, cell migration, transcription factors binding, ion transport, and the FoxO signaling pathway. Our integrative analysis of microRNA-mRNA co-profiles comprehensively characterized regulatory relationships of molecular pathways and revealed microRNAs targeting ECM-associated genes in cancer cachexia.

## Introduction

Cancer cachexia is a multifactorial syndrome characterized by an ongoing loss of skeletal muscle mass that affects up to 80% of cancer patients, depending on the cancer type (Dhanapal et al., 2011), and it represents the direct cause of at least 20% of cancer deaths (Loberg et al., 2007). Muscle wasting in cancer patients leads to a significant weight loss that affects the quality of life, response to radio- and chemotherapy, tolerance to treatment, and survival (Dewys et al., 1980; Fearon et al., 2013; Martin et al., 2013; Vaughan et al., 2013; von Haehling et al., 2016). Loss of skeletal muscle mass, the most apparent symptom of cachexia, is mainly related to metabolic dysregulation characterized by resistance to anabolic signals, increased energy expenditure, and catabolism (Porporato, 2016).

Several studies have linked cachexia to increased plasma levels of proinflammatory cytokines such as interleukin (IL) 1β, IL6, tumor necrosis factor-alpha (TNF-α), and interferon-gamma (IFN-γ) (Fearon et al., 2012). These molecules alter the ubiquitin-proteasome, insulin-like growth factor 1 (IGF1)-Akt, and autophagy-lysosome pathways, which ultimately lead to an imbalance between muscle protein synthesis and degradation (Fearon et al., 2012). However, the molecular mechanisms by which these cytokines mediate muscle wasting are not entirely understood.

Recent advances in mRNA transcriptome analysis have helped to unveil new molecular pathways, potential therapeutic targets, and biomarkers in cancer cachexia (Monitto et al., 2001; Stephens et al., 2010; Bonetto et al., 2011; Gallagher et al., 2012; Fontes-Oliveira et al., 2014; Judge et al., 2014; Shum et al., 2015; Fukawa et al., 2016). Genome-wide expression microarrays studies have also highlighted the importance of microRNAs (miRNAs) as an additional level of complex regulatory networks that post-transcriptionally control gene expression in muscle-wasting conditions (Eisenberg et al., 2007; Agarwal et al., 2013; Shen et al., 2013; Soares et al., 2014). While these studies have initially shown that global miRNA transcriptional profiles in wasting muscles seem to be peculiar to different catabolic conditions (Soares et al., 2014), it was subsequently demonstrated that the miRNA miR-29b contributes to multiple types of muscle atrophy, including those found in cardiac and cancer cachexia (Li et al., 2017; Moraes et al., 2017). Skeletal muscle miRNA transcriptome profiling using RNA sequencing (RNA-Seq) is also highly informative in cancer cachexia and has identified novel miRNAs associated with the syndrome in patients with pancreatic and colorectal cancer (Narasimhan et al., 2017). This same study also reanalyzed two independent mRNA microarrays datasets and identified that the miRNA-targeted transcripts, in a tissue-specific context, are involved in myogenesis and inflammation pathways. In a mouse model of cancer cachexia, miRNA sequencing combined with bioinformatics prediction analyses revealed that wasting muscles change the expression of miRNAs targeting transcripts that are essential for determining muscle size (Lee et al., 2017).

Although informative, these previous transcriptomic studies addressing muscle wasting in cancer cachexia did not explore different levels of gene expression regulation by integrating mRNAs and miRNAs RNA-Seq data from the same set of muscle samples. Nevertheless, miRNA-mediated regulation of gene expression in cellular networks involves complex interactions among various miRNA targets through several mechanisms (Flynt and Lai, 2008; Dragomir et al., 2018), which can be better addressed by simultaneous analysis of intrinsic interactions of biological entities across multiple omics layers. Thus, the identification of multi-omics features observed on the same set of samples provides a unique possibility for elucidating transcriptional and post-transcriptional regulatory networks during muscle wasting in cancer cachexia.

In the present study, we aimed to explore and integrate paired miRNA and mRNA co-profiles during skeletal muscle wasting in a mouse model of cancer-induced cachexia. This comprehensive analysis allowed the identification of new miRNA targets and regulatory strategies controlling gene expression in skeletal muscle atrophy. Although the atrophic muscles of cachectic mice showed high heterogeneity in the transcriptional profile, we successfully identified a set of differentially expressed genes uniformly regulated within these cachectic samples. We also characterized the regulatory relationships of molecular pathways, including miRNAs targeting extracellular matrix (ECM)-associated genes. Taken together, our results show that ECM-associated genes are dependent on inflammatory signaling and reveal miRNA-mRNA molecular networks that may play a role in the development of muscle wasting in cancer cachexia.

## Materials and Methods

### Lewis Lung Carcinoma (LLC) Model of Cancer Cachexia

To generate the LLC model of cancer cachexia, we used 8-week old, healthy C57BL/10 male mice obtained from a breeding colony, maintained by our institutional animal care facility at the São Paulo State University (UNESP, Botucatu, São Paulo, Brazil). The animals were housed in cages under a 12 h light/dark cycle with food and water *ad libitum*. Before inoculation into mice, LLC cells (ATCC^®^ CRL-1642^TM^) were cultured in Dulbecco’s modified Eagle’s medium (DMEM, Thermo Fisher Scientific, United States) supplied with 10% of fetal bovine serum (FBS, Thermo Fisher Scientific, United States) and 1% penicillin/streptomycin (Thermo Fisher Scientific, United States) and maintained in a 5% CO2, 37°C humidified incubator. After 3 days of acclimation to their environment, mice were randomly assigned into two groups. Twenty mice (LLC group) were inoculated subcutaneously with a total of 1.5 × 10^6^ LLC cells (7.5 × 10^5^ cells in 0.1 mL PBS in each flank). Ten mice (control group) were injected with equal volumes of 1X PBS.

Lewis Lung Carcinoma and control mice were weighed daily and studied 22 days after LLC cells inoculation when the LLC group had developed overt cachexia. The animals were euthanized upon anesthesia with intraperitoneal ketamine and xylazine (100 and 10 mg/kg, respectively), and tumor diameter, tumor weight, and body weight were measured. The tibialis anterior (TA), soleus (SOL), and gastrocnemius (GAS) muscles were collected, weighed, and then snapped frozen in liquid nitrogen for further analyses. These muscles were chosen based on their fiber composition: TA muscle has a higher proportion of glycolytic fast-twitch fibers, SOL muscle has a higher proportion of oxidative slow-twitch fibers, and the GAS muscle, which comprises both fiber types.

The study was performed following the guidelines of the Control of Animal Experimentation and Ethical Principles in Animal Research (CONCEA - National Council for Control of Experimental Animals), under the approved protocol n° 702, emitted by the Institute of Bioscience of Botucatu Ethics Committee on Animal Use, from the São Paulo State University (UNESP, Brazil).

### Total RNA Isolation

RNA extraction was performed using the TRIZOL reagent (Thermo Fisher Scientific, United States), according to the manufacturer’s instructions. RNA was quantitated by spectrophotometry (NanoVue; GE Healthcare Life Sciences, United States), and its integrity was ensured by obtaining RNA Integrity Number (RIN) > 8 (Agilent 2100 Bioanalyzer; Agilent Technologies, Germany). RNA samples were treated with DNA Free Kit (Thermo Fisher Scientific, United States) to remove genomic DNA contamination.

### Preparation and Processing of mRNA-Seq Libraries

We randomly selected samples (four control and six LLC mice) to construct the RNA sequencing libraries with the TruSeq Stranded Total RNA Sample Prep Kits (Illumina, United States), using 1000 ng of total RNA. These six LLC mice are those that survived the entire period of the experiment (22 days). Samples were indexed with adaptors and submitted for paired-end 2 × 100-bp sequencing using a HiSeq 2000 instrument (Illumina, United States). Sequencing was performed on ten RNA samples in one lane of the flow cell, following the manufacturer’s instructions. The lane produced ∼ 600 million raw paired reads. The data output in the fastq file format contained sequence information, including the sequencing quality (Phred quality score). Average Phred scores ≥20 per position were used for alignment.

### Preparation and Processing of miRNA-Seq Libraries

The TruSeq Small RNA Sample Preparation kit (Illumina, United States) was used to prepare the miRNA-Seq libraries for the same set of samples used for mRNA-Seq, following the manufacturer’s instructions. miRNA libraries were 50 bp single-end sequenced using a HiSeq2000 instrument (Illumina, United States). Sequencing was performed on ten RNA samples in one lane of the flow cell.

### Read Alignment and Differential Gene Expression Analysis

Paired-end reads for mRNA were mapped to the mm10 genome using TopHat2 (Kim et al., 2013) with the following options: –mate-inner-dist 200 –mate-std-dev 100 –no-novel-juncs –min-intron-length 40. Single-end reads for miRNA were mapped to the miRBase version 21 using Bowtie (Langmead et al., 2009) with the following options: -n 0 -l 8 -a –best –strata –phred33-quals. Counts for RefSeq genes were obtained using HTSeq (Anders et al., 2015) with the default settings, and DESeq2 (version 1.4) (Love et al., 2014) was used to normalize expression counts. Fold change was calculated as the ratio of normalized counts for each sample against the average of the reference group. Genes were considered differentially expressed if | fold change| (FC) ≥ 1.5, and *p*-values ≤ 0.05. For assess mRNA transcript abundance, reads were converted to reads per thousand base pairs peak per million mapped reads (RPKM). For assessing miRNA abundance, the reads were converted to counts per million mapped reads (CPM). Finally, we used dot plots to demonstrate the expression of selected genes either previously associated with muscle atrophy in cachexia or that we hypothesized may be associated with muscle atrophy, based on the literature.

### Clustering Analysis of the RNAseq Expression Data

We standardized the normalized counts of each gene and applied k-means clustering using Euclidian distance and with random initialization and 10000 executions, and finally selecting four clusters.

### Motif Analysis

Pscan web interface (Zambelli et al., 2009)^1^ was used to detect DNA motifs overrepresented in the promoter of the differentially expressed genes. Gene promoters were considering between nucleotides −300 and +50 relative to the Transcription Start Site (TSS). Significance was tested against CpG-content-matched promoters as background. Binding sites were considered significantly overrepresented when the *p*-value <0.01.

### Gene Ontology (GO) Enrichment Analysis

Gene Ontology enrichment was performed using the ClueGO Cytoscape plugin (Bindea et al., 2009), using a hypergeometric test with a Benjamini-Hochberg False Discovery Rate correction (Benjamini and Hochberg, 1995). A *p*-value cut-off of 0.05 was used to identify enriched terms.

### miRNA Target Prediction

Candidate miRNA-mRNA targets relationships were predicted by at least one or more of the following target prediction algorithms (union set) extracted from mirDB (Wang, 2008), TargetScan 5.1 (conservation and non-conservation sites) (Garcia et al., 2011), DIANA-microT (Maragkakis et al., 2009), and PicTar (4-way, and 5-way) (Krek et al., 2005). Additionally, we used validated targets deposited in miRTarBase (Hsu et al., 2011). We also filtered our data using differentially expressed genes (mRNA and miRNA) identified by RNA-Seq, considering that mRNA and miRNA expression levels should be inversely correlated.

### Interaction Network

Based on the differentially expressed genes, protein-protein interaction networks were generated using the STRING database (Snel et al., 2000; Szklarczyk et al., 2017)^2^, which also detects functional interactions among the corresponding genes. Network visualization was performed using the open-source software platform Cytoscape (Shannon et al., 2003) v. 3.6.1^3^.

### Picrosirius Red Staining

Cryostat transverse sections of the TA muscle (10 μm thick) were collected from control and LLC tumor-bearing mice. Collected samples were placed on the same slide to minimize staining differences; sections were incubated with a saturated picric acid solution followed by Picrosirius red (0.1% Sirius red in saturated picric acid) for 3 min, dehydrated, and mounted in Permount. Eight color pictures per sample were captured using a light microscope (Olympus, Japan). The light intensity parameters used were the same for all samples. Picrosirius staining areas were assessed using the Image J software. As previously described, picrosirius staining areas were normalized for cell density to account for individual fiber atrophy (Kirby et al., 2005). To quantify muscle tissue disorganization, we employed fractal dimension analysis by binarizing photographs using ImageJ software, as previously described (Pacagnelli et al., 2016). Briefly, the fractal dimension was estimated using the box-counting tool (ImageJ software), which quantifies pixel distribution in the space without considering image texture. The fractal dimension value is expressed from 0 to 2, where values close to 2 represent higher tissue disorganization.

### Western Blotting Analysis

Muscle proteins were extracted using Tris-Triton buffer (10 mM Tris pH 7.4, 100 mM NaCl, 1 mM EDTA, 1 mM EGTA, 1% Triton X-100, 10% glycerol, 0.1% SDS, 0.5% deoxycholate) containing Protease Inhibitor Cocktail (Sigma-Aldrich, United States) and quantified by the Bradford method (Bradford, 1976). Subsequently, the Lammeli buffer (Sigma-Aldrich, United States) was added to each sample and boiled at 100°C for 10 min. Proteins were subjected to SDS-PAGE on 10% polyacrylamide gels. After electrophoresis, proteins were electrotransferred to nitrocellulose membranes (Bio-Rad, United States) for 2 h at 120V. The membranes were blocked with 5% non-fat dry milk diluted in TBS-Tween for 2 h, and then incubated overnight at 4°C with collagen I (1:100 dilution, sc-25974, Santa Cruz, CA, United States) or β-actin (1:1000 dilution, sc-81178, Santa Cruz, CA, United States) antibodies. Secondary antibodies conjugated with horseradish peroxidase (HRP) and ECL chemiluminescent detection (GE Healthcare, United States) system were used for visualization of the blots in ImageQuantTM LAS 400 (GE Healthcare, United States). We quantified the blots by densitometry using ImageJ software, and collagen *I* values were normalized to β-actin.

### Statistical Analysis

Data are expressed as mean ± standard deviation (SD). Statistical analysis was performed using the GraphPad Prisma software v 6.07 (GraphPad Software, Inc., United States). For all statistical analyses not described elsewhere, Student’s *t*-test was applied to compare the groups. Statistical significance was considered achieved when the *p*-value was <0.05.

## Results

### Lewis Lung Carcinoma Cells Induced Cachexia in Mice

As expected, all mice subcutaneously inoculated with Lewis Lung Carcinoma (LLC) cells developed cancer cachexia (LLC group) compared to mice injected with PBS (control group). The tumor was detected by palpation after 7 days of cell injection in LLC, after 15 days, the tumor site was visually identified as a skin projection; and after 22 days, when the animals were euthanized, tumor mass was observed under the skin. After euthanasia, the surgically exposed tumor was solid, vascularized, roughly spherical, measuring ∼2 cm in diameter, and weighing ∼4 g (Supplementary Figures S1A,B).

**FIGURE 1:**
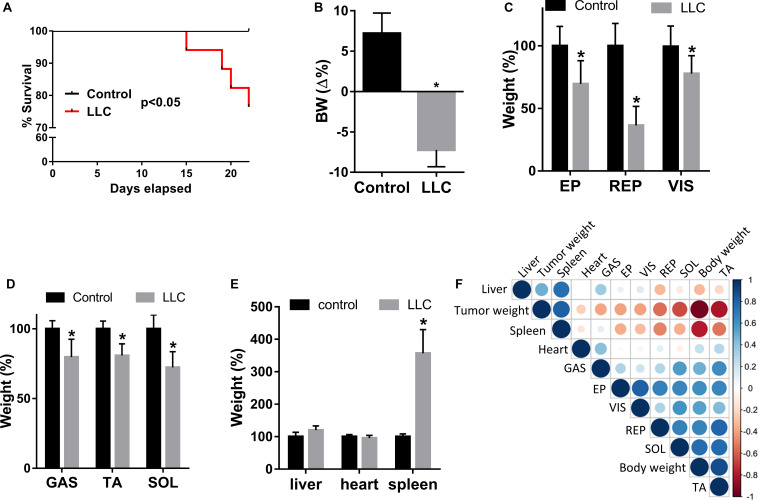
Lewis Lung Cancer (LLC) cells induce cachexia in mice. **(A)** Kaplan-Meier survival curves of control and tumor-bearing mice (LLC) groups. **(B)** Body weight (BW = Δ%), defined as total BW - tumor weight, and reported as a percentage of the initial BW. **(C)** Epidydimal (EP), retroperitoneal (RP), and visceral (VIS) fat weight loss in LLC respective to control. **(D)** Gastrocnemius (GAS), tibialis anterior (TA), and soleus (SOL) muscle weight loss in LLC respective to control. **(E)** Liver, heart, and spleen weight in LLC respective to control. **(F)** Triangular heatmap representing pairwise Pearson correlation of the different morpho-anatomical data; blue and red dots represent positive and negative correlations, respectively. Data are expressed as mean ± SD; control (*n* = 10) and LLC (*n* = 20). *p < 0.05: statistical significance compared to the control group (two-tailed *t*-test).

Although the LLC cell line is highly tumorigenic, metastases were not visually identified. Five out of twenty mice died during the experiment (25%) (Figure 1A). Consistent with cachexia syndrome, the LLC group exhibited 14.4% body weight (BW) loss after 22 days of LLC cell injection compared to the control group (Figure 1B and Supplementary Table S1). This BW reduction was associated with a loss of adipose tissue (Figure 1C and Supplementary Table S1) and skeletal muscle mass (Figure 1D and Supplementary Table S1). Cachexia was further confirmed by splenomegaly (Figure 1E, Supplementary Table S1, and Supplementary Figure S1C). Finally, among the studied muscles – tibialis anterior (TA), soleus (SOL), and gastrocnemius (GAS) – we selected TA for further analysis because its weight presented strong correlations between tumor weight (inverse correlation) and BW (positive correlation) (Figure 1F).

### Comprehensive Transcriptome Characterization of Muscle Wasting

**FIGURE 2:**
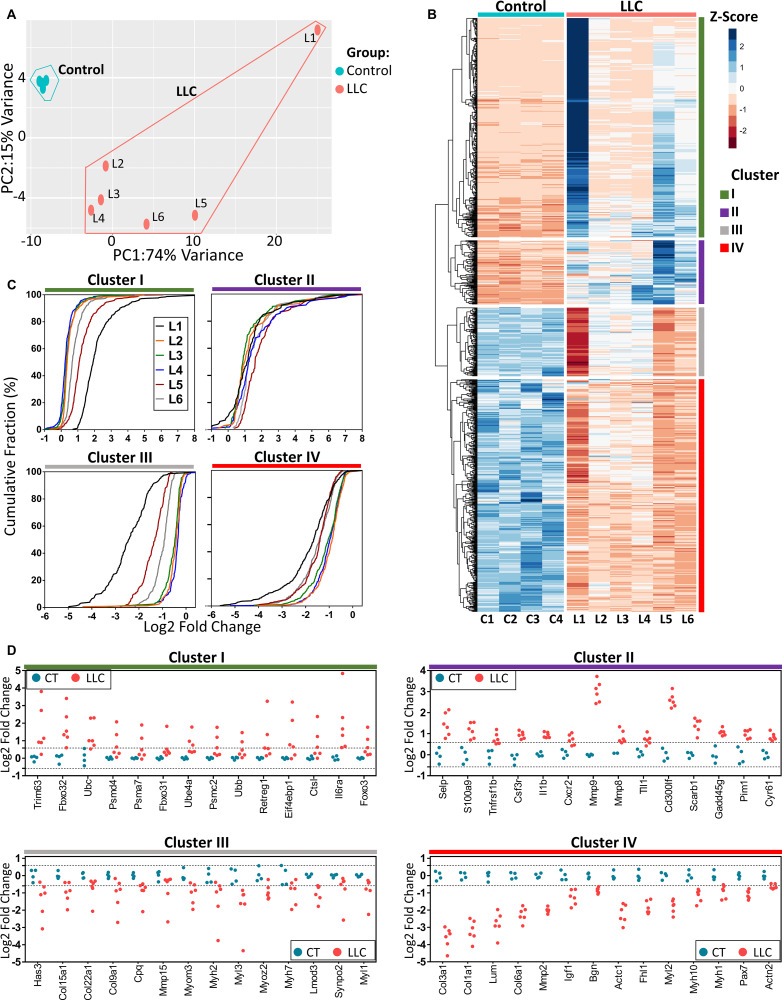
Comprehensive transcriptome characterization of muscle wasting revealed differential gene expression stability in cancer cachexia. **(A)** Principal component analysis of the gene expression data of control and tumor-bearing mice (LLC) groups. The percentage of the variance of each principal component (PC1 and PC2) for control (C1–C4) and LLC samples (L1–L6). **(B)** Heatmap of 1008 Z-score normalized differentially expressed genes of control (C1–C4) and LLC samples (L1–L6) by unsupervised hierarchical clustering analysis identified the clusters I (*n* = 386), II (*n* = 101), III (*n* = 157), and IV (*n* = 364). Down-regulated and up-regulated genes with absolute values of fold-change > 1.5 and FDR < 0.05 (Wald statistics) are shown in red and blue dots, respectively. **(C)** Cumulative frequency distribution of the differentially expressed genes (log2-fold change, *x*-axis) for LLC (L1–L6) vs. control samples, indicated as a percentage (%, *y*-axis) for each cluster (I to IV) identified in panel **(B)**. **(D)** Dot plots of differentially expressed genes selected for each cluster (I to IV) identified in panel **(B)** to demonstrate the range in expression variability across genes within LLC samples. Light blue and pink dots represent control and LLC samples, respectively. These genes were identified as either previously associated with muscle atrophy in cachexia or that we hypothesized may be associated with muscle atrophy, based on the literature. The threshold for up- and down-regulated (|fold change| ≥ 1.5) are indicated by dashed lines.

Transcriptome analysis of TA muscle revealed 11,436 genes out of the nearly 45,000 mouse RefSeq genes. Principal Component Analysis (PCA) was able to discriminate LLC and control samples (Figure 2A). High heterogeneity in mRNA profiles of the LLC group was evidenced by spatial dispersion. We found 1008 differentially expressed genes (DEGs), of which 487 and 521 were up- and down-regulated, respectively (Supplementary Table S2). We validated by RT-qPCR the expression of some genes involved in myogenesis, sarcomere, proteasome, and ECM (Supplementary Figure S2). Unsupervised hierarchical clustering analysis showed that DEGs were organized in four clusters (I–IV), according to their direction and variability of expression within LLC samples (Figure 2B): clusters I (*n* = 386) and II (*n* = 101) contain up-regulated genes, while clusters III (*n* = 157) and IV (*n* = 364) include down-regulated genes. Considering the gene expression variability within LLC samples, clusters II and IV contain genes that are uniformly regulated (fold change% CV: 32.62 ± 15.50 and 32.49 ± 15.90, respectively Figure 2C); whereas clusters I and III contain genes with high variability in expression levels among LLC samples (fold change% CV: 65.60 ± 38.82 and 54.97 ± 15.42, respectively, Figure 2C).

Next, we explored the identity of the genes found within the clusters. Cluster I include up-regulated genes that have variable expression. The genes in cluster I are associated with the proteasome complex (e.g., *Trim63*, *Fbxo32*, *Ubc*, *Ubb*, *Psmd4*, *Psma7*, *Psmc2*, *Fbxo31*, and *Ube4a*), autophagosome (e.g., *Ctsl* and *Retreg1*), and the translation inhibitor *Eif4ebp1* (Figure 2D and Supplementary Figure S3A). Remarkably, cluster I includes the interleukin-6 receptor (*Il6ra*), which presented a 5-log range in expression variability within LLC samples (Figure 2D). Cluster III contains down-regulated genes that have variable expression; this cluster includes genes associated with ECM (e.g., *Has3*, *Col15a1*, *Col22a1*, *Col9a1*, *Cpq*, and *Mmp15*) and muscle metabolism and contraction (e.g., *Myom3*, *Myh2*, *Myl3*, *Myoz2*, *Myh7*, *Lmod3*, *Synpo2*, and *Myl1*) (Figure 2D and Supplementary Figure S3A). Cluster II comprises up-regulated genes that are uniformly regulated within LLC samples, which are associated with the immune system (e.g., *Selp*, *S100a9*, *Il1b*, *Cxcr2*, and *Csf3r*), ECM organization (e.g., *Mmp9*, *Mmp8*, and *TLL1*), and apoptotic process (e.g., *Cd300lf*, *Scarn1*, *Lcn2*, *Gadd45g*, and *Chac1*) (Figure 2D and Supplementary Figure S3A). Cluster IV contains down-regulated genes that are uniformly regulated within LLC samples, which are associated with ECM and sarcomere (Figure 2D and Supplementary Figure S3A). Remarkably, both cluster III and IV contain genes related to the ECM (collagens) and sarcomere (myosins), but with differences in gene expression stability (low and high, respectively) within LLC samples. Considering the variability in gene expression profiles within the LLC samples, we determined a reduced set of DEGs able to differentiate cachectic and control groups. For this, we use Euclidian distances, and we found that samples L2, L3, and L4 are part of a cluster that we call cluster A which is the closest to the control group (Supplementary Figure S3B). Next, we determined the number of DEGs between the subgroup of samples. We found 443 DEGs that were sufficient to effectively segregate both LLC and control groups (Supplementary Figure S3D), presenting high intragroup stability (fold change% CV: 28.95 ± 10.02 and 27.30 ± 11.06, for up- and down-regulated genes, respectively) (Supplementary Figures S3E,F). This set of DEGs was determined using samples from cluster A and comprises genes associated with the ECM, proteolysis, and inflammatory response (Supplementary Figure S3G). This set of 443 genes represents a reduced number of deregulated genes in all cachectic muscle samples, regardless of the variability in the gene expression within LLC samples.

### Relevant Genes and Regulatory Pathways Associated With Muscle Wasting in Cancer Cachexia

**FIGURE 3:**
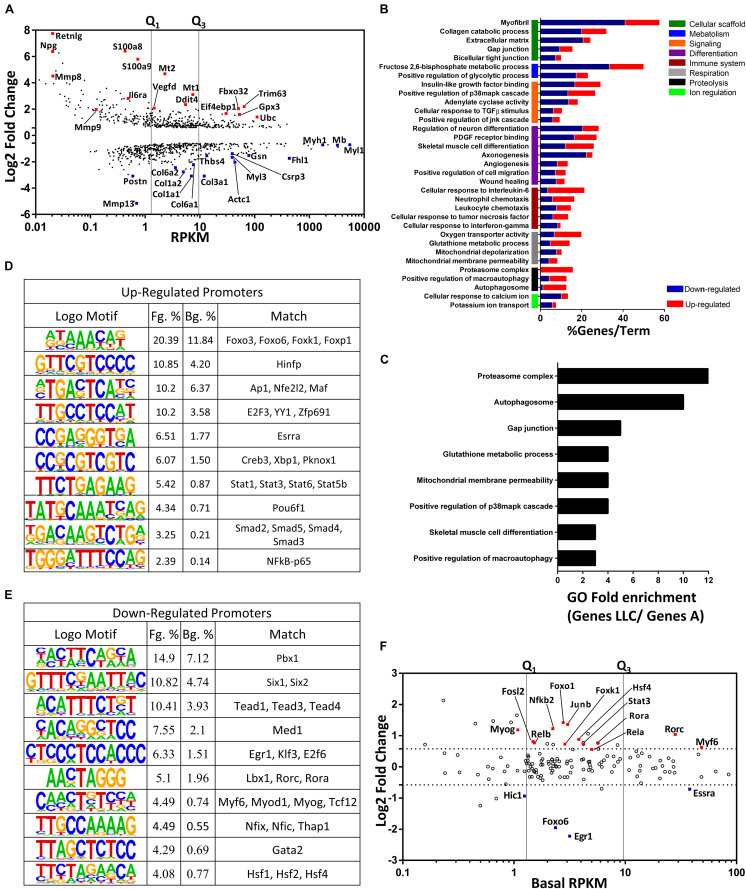
Relevant genes and regulatory pathways associated with muscle wasting in cancer cachexia. **(A)** Scatterplot comparing abundance (RPKMs, *x*-axis) and their degree of expression (log2-fold change, *y*-axis). Red and blue dots represent up- and down-regulated genes (fold-change > 1.5 and FDR < 0.05; Wald test), respectively. These genes were either previously associated with muscle atrophy in cachexia or that we hypothesized may be associated with muscle atrophy, based on the literature. Vertical lines represent thresholds for low, medium, and high abundance genes as defined by quartiles (Q1 and Q3) **(B)** Gene-term enrichment analysis of differentially expressed genes (DEGs) in the tibialis anterior of Lewis Lung Cancer (LLC) tumor-bearing mice showing the top canonical pathways. The colored horizontal bars represent the percentage of genes presented in the dataset compared to the total number of genes in each term (% Genes/Term). The fraction of up- and down-regulated genes (horizontal bars) in each term are shown in red and blue, respectively. The vertical colored bars (*y*-axis) represent major gene terms modules. **(C)** Gene ontology analysis of DEGs from LLC vs. LLC subgroup A samples. Each horizontal black bar represents the ontology term fold enrichment compared to the total number of genes in each term. *De novo* motif analysis was performed on promoters (–300 and +50 relative to Transcription Start Site, TSS) of up- **(D)** and down-regulated **(E)** genes. Motifs were compared using the transcription factor JASPAR database to determine the closest annotated matches. Percentage (%) represents a fraction of foreground (Fg) and background (Bg) sequences that contain at least the occurrence of one motif. **(F)** Scatterplot comparing abundance (Basal RPKMs, *x*-axis) and their degree of expression (log2-fold change, *y*-axis). Each dot represents a differentially expressed genes (DEGs; fold-change > 1.5 and FDR < 0.05; Wald test) encoding for transcription factors (red and blue dots, up- and down-regulated genes, respectively). Vertical lines represent thresholds for low, medium, and high abundance genes, as defined by quartiles (Q1 and Q3).

We considered as biologically relevant for the muscle wasting those most abundant transcripts with the highest degree of regulation. Initially, we used a scatter plot that integrated the degree of regulation (fold change; FC) and abundance (Reads Per Kilobase Million; RPKM) of all transcripts (Figure 3A). Abundant transcripts presented subtle changes in gene expression when compared to rare transcripts. Notably, the up-regulated genes *Trim63*, *Fbxo32*, and *Ubc* - associated with the proteasomal degradation pathway - presented the highest abundance and degree of change in expression (Figure 3A). Additionally, we identified the up-regulated genes *Ddit4* and *Eif4ebp1* (Figure 3A), which have been previously implicated in protein synthesis during skeletal muscle atrophy (Bodine et al., 2001). We also found up-regulation in the expression of the antioxidant genes *Gpx3*, *Mt1*, and *Mt2* (Figure 3A), which have been described with a role in muscle repair in atrophic conditions (Nuoc et al., 2017; Summermatter et al., 2017). We identified the down-regulation of the sarcomere genes *Myl1*, *Myh1*, *Fhl1*, *Gsn*, *Myl3*, and *Actc1* in cachectic muscle samples (Figure 3A). Interestingly, we found that the muscle-specific myoglobin (*Mb*) transcript was highly abundant and is down-regulated cachexia (Figure 3A). Also, a high degree of down-regulation of the ECM genes *Col3a1*, *Thbs4*, *Col6a1*, *Col1a1*, *Col1a2*, and *Col6a2* was identified. Finally, we detected low abundance transcripts with a high degree of regulation, including Il6ra and the ECM remodeling genes *Mmp9* and *Mmp8* (Figure 3A).

Gene ontology analysis revealed 33 terms that were clustered in eight main modules that are relevant to muscle wasting, which include genes associated with the scaffold, cellular metabolism, cellular signaling, cellular differentiation, cellular immune system process, cellular respiration, proteolysis, and ion regulation (Figure 3B and Supplementary Table S3). Notably, some novelties were found, such as the negative regulation of cell junctions (e.g., gap and tight junctions), carbohydrate metabolism (e.g., glycolytic process), cell differentiation (e.g., axonogenesis, angiogenesis, and PDGF signaling), and positive regulation of the immune cell system (e.g., neutrophil and leukocyte chemotaxis) (Figure 3B). Moreover, previously cachexia-associated terms such as negative regulation in the sarcomere, cell migration, and ECM genes, as well as positive regulation of genes involved in the proteasome complex, autophagy, IL-6 signaling, and cell differentiation were detected (Figure 3B). We also identified the percentage of up- and down-regulated genes in each ontology term (Figure 3B). Consistent with the atrophic phenotype, this analysis demonstrated that all deregulated genes related to proteasome complex were up-regulated, while most of the deregulated genes related to myofibril and ECM were down-regulated (Figure 3B). Considering the transcriptome variability across the LLC samples, we also asked which pathways were enriched in LLC transcriptome when compared with the reduced set of genes enriched explicitly in the LLC subgroup A. This analysis confirmed changes in the expression of genes associated with protein degradation such as proteasome complex, autophagosomes, and macroautophagy (Figure 3C).

### Transcriptional Factors Motifs Enriched During Muscle Wasting in Cancer Cachexia

The transcriptional profile can provide a step toward the identification of key transcription factors that regulate gene expression. We performed an enrichment analysis of transcriptional motifs in the promoter sequences of 1008 differentially expressed genes (Figures 3D,E). The promoters of the up-regulated genes revealed motif enrichment for the Forkhead transcription factor (FoxO) (Figure 3D). However, when we analyzed the changes in expression and abundance of FoxO family members genes, only *FoxO1* and *FoxO6* were up- and down-regulated, respectively (Figure 3F). The promoters of the up-regulated genes also revealed binding sites for transcriptional factors within the NF-κB and STAT families (Figure 3D). Additionally, components of the NF-κB and STAT families were up-regulated: *Rela*, *RelB*, *Nfkb2*, and *Stat3* (Figure 3F). We found enrichment of the AP-1 transcription factor, as well as the up-regulation of *Junb* and *Fosl2*, which are translated into proteins that constitute the AP-1 heterodimer (Figure 3F). We also found enrichment of other transcriptional factors without a change in their expression (Figures 3D,F); among these are transcription factors related to cell cycle regulation (*E2f3*, *Yy1*, and *Creb3*), unfolding protein response (*Xbp1*), and the SMAD family (*Smad2*, *Smad3*, *Smad4*, and *Smad5*).

Interestingly, promoters of down-regulated genes also revealed motif enrichment of transcriptional factors related to myogenesis (*Myf6*, *Myod1*, *Myog*, *Tcf12*, *Pbx1*, *lbx1*, *Nfix*, and *Nfic*), lipid homeostasis (*Rora* and *Rorc*), energy metabolism (*Med1*), and muscle fiber-type specification (*Six1*, *Six2*, *Tead1*, *Tead3*, *Tead4*, *Egr1*, *Klf3*, *Hsf1*, *Hsf2*, and *Hsf4*) (Figure 3E). However, only genes coding for the transcription factors *Myf6*, *Myog*, *Rorc*, *Rora*, and *Egr1* changed their expression (Figure 3F).

### miRNAs Associated With Muscle Wasting in Cancer Cachexia

**FIGURE 4:**
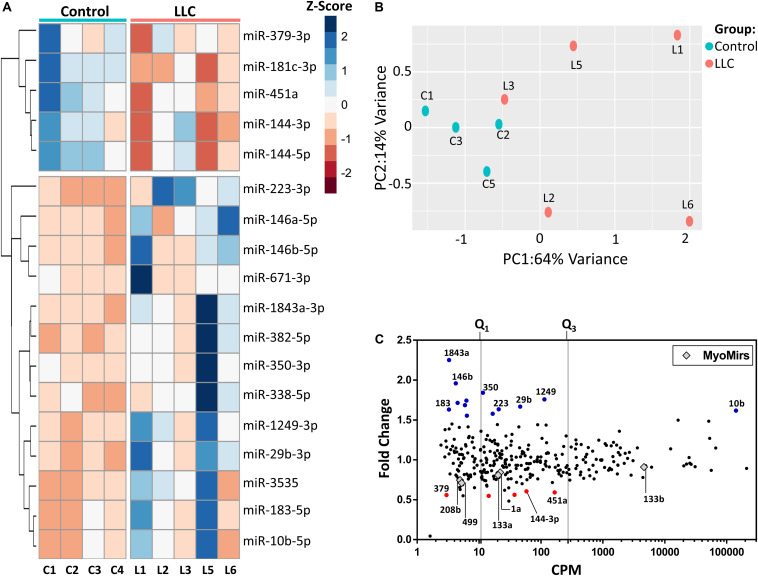
Differentially expressed miRNAs in cancer cachexia. **(A)** Principal component analysis of gene expression data of control and tumor-bearing (LLC) mice. The percentage of the variance of each principal component (PC1 and PC2) for the control (C1–C4) and LLC (L1–L3, L5–L6) samples. Sample L4 did not pass the quality filters, and it was removed from the analysis **(B)** Heatmap of 18 Z-score normalized differentially expressed miRNAs of control (C1–C4) and LLC (L1–L3, L5–L6) samples by unsupervised hierarchical clustering analysis. Down-regulated and up-regulated miRNAs with absolute values of fold-change > 1.5 and FDR < 0.05 (Wald statistics) are shown in red and blue, respectively. **(C)** Scatterplot comparing abundance (Counts per Million, CPM; *x*-axis) and their degree of expression (log2-fold change, *y*-axis). Each dot represents differentially expressed miRNAs (fold-change > 1.5 and FDR < 0.05; Wald test), and red and blue dots (up- and down-regulated miRNAs, respectively) highlight potentially relevant miRNAs associated with muscle wasting. Gray diamonds represent the muscle-specific miRNAs (MyomiRs). Vertical lines represent thresholds for genes with low, medium, and high abundance, as defined by quartiles (Q1 and Q3).

Out of 1915 mature miRNAs, 302 were expressed in skeletal muscle (mapped reads >32 in at least one of the sequenced samples). Eighteen miRNAs were differentially expressed (FDR ≤ 0.05 and |fold change| ≥ 1.5) in muscle wasting during cancer cachexia in comparison to controls (13 up and five down-regulated, Figure 4A). PCA and clustering analysis showed that these 18 miRNAs were poorly clustered samples according to their experimental groups (Figure 4B) when compared to the clear clustering found in PCA of the mRNA expression data (Figures 2A,B). Furthermore, 44% of differentially expressed miRNAs in LLC muscle samples were expressed at low levels and the degree of regulation (Figure 4C). Notably, miR-10b-5p was regulated at high levels in the atrophying muscles (Figure 4C). The differentially expressed miRNAs also included miR-29b-3p, miR-146a-5p, miR-146b-5p, and miR-181c-3p, which have been previously studied in the skeletal muscle context (Naguibneva et al., 2006; Li et al., 2017; Sun et al., 2017). Interestingly, the MyomiRs mir-208a, mir-208b, mir-499, miR-133a, miR-133b, and miR-1 were not differentially expressed in the LLC group.

### Integrative Analysis Revealed a Set of ECM mRNAs Regulated by miRNAs in Cancer Cachexia

**FIGURE 5:**
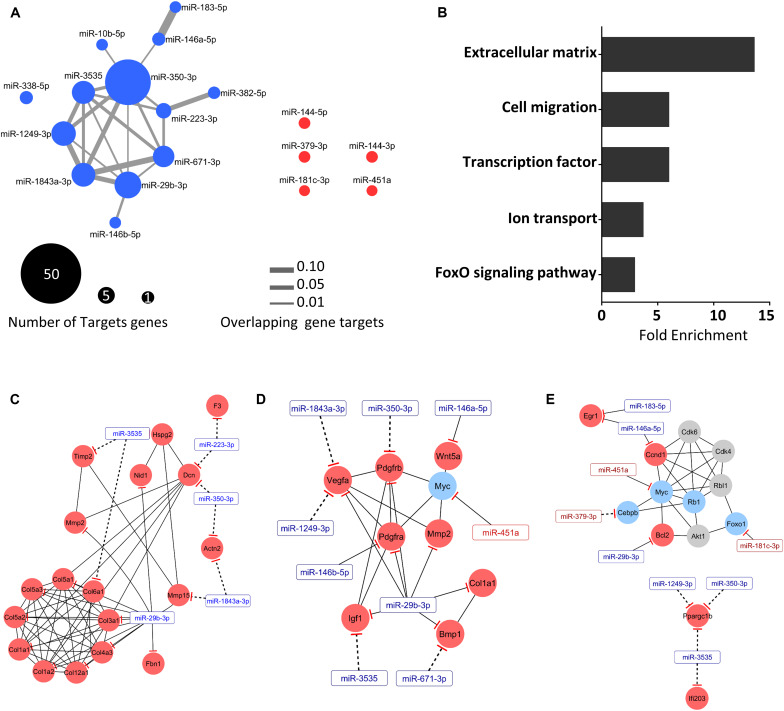
Integrative analysis revealed a set of extracellular matrix mRNAs regulated by miRNAs. **(A)** The network generated is consisted of 171 interactions between 18 miRNAs and 131 target genes transcripts. Up- and down-regulated miRNAs (fold-change > 1.5 and FDR < 0.05; Wald test) are represented by blue and red nodes, respectively. Node size indicates the number of miRNA-target gene transcripts, and the gray edge width denotes overlapping miRNA-target gene transcripts measured by the Jaccard coefficient (JC). **(B)** Gene ontology analysis of the mRNAs predicted and validated as regulated by miRNAs. Each horizontal black bar represents the ontology term fold enrichment compared to the total number of genes in each term. Regulatory network displaying predicted (dashed lines) and validated (solid lines) interactions between the miRNAs (rectangle) and target mRNAs (circles), and physical and pathway protein-protein interactions (solid lines) for extracellular matrix **(C)**, cell migration **(D)**, and transcription factors **(E)**. Up- and down-regulated miRNAs (fold-change > 1.5 and FDR < 0.05; Wald test) are represented by blue and red nodes, respectively. Gray nodes represent non-regulated genes.

To improve the accuracy of our *in silico* mRNA target prediction used to identify potential mRNA targets of the differentially expressed miRNAs, we considered opposite directions of deregulated expression between miRNA and target mRNAs in the same set of samples. We found a network with 171 interactions between 18 miRNAs and 131 target genes (Figure 5A). This analysis revealed that the upregulated miRNA miR-350-3p has a higher number of target genes (*n* = 47). Interestingly, miR-29b-3p presented 22 potential targets, including many genes that encode proteins related to the ECM. Additionally, we found that repressed miRNAs do not share target genes. Furthermore, some genes such as *Map2k6*, *Ptpn3*, *Mettl21c*, *Plxdc2*, *Ppargc1b*, *Rgs5*, and *Vegfa* were found to be co-regulated by up to three upregulated miRNAs (Supplementary Table S4).

Based on the integrative miRNA-mRNA analysis, we identified enriched pathways for deregulated genes targeted by differentially expressed miRNAs (Figure 5B). Gene ontology analysis revealed miRNA interactions affecting genes regulating mainly the ECM, but also cell migration, transcription factor binding, ion transport, and FoxO signaling. To elucidate the functions of these complex interactions between mRNAs and miRNAs in cancer cachexia, we constructed a regulatory network displaying predicted and validated interactions between the miRNAs and target mRNAs, considering physical and pathway protein-protein interactions. We found sub-networks such as those related to ECM organization (Figure 5C), cell migration (Figure 5D), and transcription factors (Figure 5E). The ECM organization network (Figure 5C) contains a set of nine collagen genes, including validated targets of the miR-29b-3p. Furthermore, we found predicted interactions for miR-1843a-3p, miR-350-3p, miR-223-p, and miR-3535 with ECM components such as *Col6a1*, *Timp2*, *Mmp15*, *Dcn*, and *Actn2*. These identified networks share the miRNAs mir-29b-3p, mir350-3p, and miR-3535, suggesting a pleiotropic effect of these miRNAs on the ECM of atrophying muscles in cancer cachexia.

### ECM Remodeling in Cancer Cachexia

**FIGURE 6:**
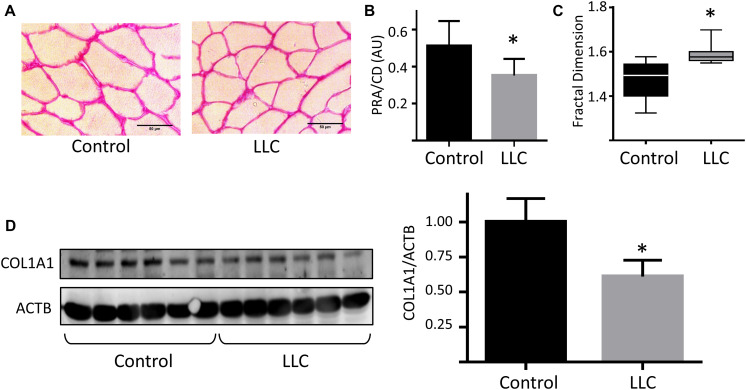
Extracellular matrix remodeling in skeletal muscle during cancer cachexia. **(A)** Histological sections of tibialis anterior (TA) muscle stained with Picrosirius-red staining at 20× magnification of control and LLC tumor-bearing mice muscles. **(B)** Quantitative analysis of Picrosirius-red staining areas (PRA) normalized by cell density (CD). **(C)** Fractal dimension analysis of TA Picrosirius-red staining areas of control and LLC tumor-bearing mice muscle. **(D)** Protein levels of Col1a1 in TA muscle of control and LLC tumor-bearing mice; blots were normalized by α–actin protein levels. ^∗^*p* < 0.05: statistical significance compared to the control group (two-tailed *t*-test).

Together, our results point out to a crucial role of ECM remodeling in skeletal muscle atrophy in cancer cachexia. TA muscle cross-sectional area stained with Picrosirius red in LLC tumor-bearing mice presented reduced total collagen deposition (Figures 6A,B) and ECM disarrangement (Figure 6C), as revealed by picrosirius red staining area and fractal dimension analysis, respectively. The reduction of ECM was further confirmed by the reduced protein levels of collagen alpha-1 type I collagen (COL1A1), one of the main structural components of the ECM in skeletal muscle tissues (Figure 6D). We also found ECM genes deregulated in the gastrocnemius and soleus muscles (Supplementary Figure S4).

## Discussion

Despite advances in the study of cancer cachexia, its pathogenesis is complex and remains incompletely understood (Baracos et al., 2018). Thus, it is necessary to identify signaling pathways as well as transcriptional and post-transcriptional events underlying skeletal muscle atrophy in this condition. We used paired microRNA-mRNA co-profiles in wasting muscles of cachectic mice, which unveiled ECM remodeling events potentially regulated by miRNAs. Although our transcriptomic analysis demonstrated a high heterogeneity in mRNA profiles of cachectic mice, we successfully identified a reduced number of differentially expressed genes that were uniformly regulated with low variability within cachectic samples. Thus, in addition to the well-known down-regulation of sarcomere proteins genes, other genes encoding ECM structural proteins that are potentially regulated by miRNAs may contribute to the development of skeletal muscle wasting in cancer cachexia.

Our RNAseq data significantly expands previous genome-wide studies in cancer cachexia by integrating mRNA and microRNA transcriptome profiling from the same set of muscle samples. This strategy allowed us to identify miRNA targets with higher accuracy. Moreover, we used a high number of biological replicates (six cachectic and four controls), which added higher precision and sensitivity for the identification of transcriptional and post-transcriptional events. Our transcriptomic data reliably differentiated muscle samples from cachectic and control mice. Notably, inter-individual variations in mRNA expression were evidenced in cachectic mice, which may be linked to individual genetic factors and stochastic tumor growth events that may determine the development and progression of muscle wasting. Studies in rodent models show heterogeneity in the occurrence of cachexia (Matsuyama et al., 2015; Norden et al., 2015). This characteristic is also found in human neoplasms, which shows variability in the prevalence and severity of cachexia among patients with the same diagnosis and cancer stage (Prado et al., 2013; Baracos et al., 2018). Importantly, we found some genes commonly associated with cachexia, which presented high variability in expression levels among cachectic mice. These data may help to explain the variability in the prevalence and severity of the syndrome. For example, the highly variable up-regulated genes are related to protein catabolism (e.g., *Trim63* and *Fbxo32*, Figure 2), while the highly variable down-regulated genes are mainly associated with sarcomere and ECM. This variability in the expression profile of these sets of genes within LLC samples may help to explain why protein catabolism genes in human studies have failed to recapitulate the findings from murine models (Gallagher et al., 2012; Johns et al., 2017). This variability in gene expression, also known as noise, has been described as an essential feature of any biological system (Eldar and Elowitz, 2010), and previous gene expression studies have been able to classify different states of diseases based on this variability (Mar et al., 2011; Ecker et al., 2015; Zhang et al., 2015; Guan et al., 2016).

To generate a complete picture of transcriptome content and dynamics, we explored differential expression data to identify transcriptional factor-binding motifs. Our motif analysis for up-regulated genes identified enrichment for several transcription factors, including FoxO, NF-κB, AP-1, Stat3, and Smad. These factors have already been described in the regulation of muscle atrophy by activating genes related to the proteolytic ubiquitin-proteasome, autophagy-lysosomal, metabolic adaptation, myogenesis, differentiation, and immune-modulation (Cai et al., 2004; Costelli et al., 2005; Choi et al., 2012; Gerstein et al., 2012; Zhang et al., 2013; Chen et al., 2017). Similarly, motif analysis also showed that down-regulated genes were associated with myogenic transcription factors (Berkes et al., 2004; Watanabe et al., 2007; Rossi et al., 2016), metabolism (Chen et al., 2010), fiber transition (Grifone et al., 2004; Tsika et al., 2008), and muscle contraction (Pacini et al., 2013). Most importantly, our motif analysis revealed transcription factors that have not yet been reported or identified previously in cancer cachexia. These factors are related to cell cycle and myogenesis (*E2f3*, *Yy1*, and *Creb3*) (Asp et al., 2009; An et al., 2014; Zhou et al., 2015), unfolding protein response (Xbp1) (Jheng et al., 2018), and muscle fiber metabolism (ESRRA) (LaBarge et al., 2014). Also, we identified increased expression of the myogenic regulatory factors *Myf6* and *Myog*. Interestingly, it has been demonstrated that the over-expression of Myf6 inhibits the transcription of sarcomeric proteins by inhibiting Mef2 (Moretti et al., 2016). On the other hand, Myog induces the expression of the atrogenes *Trim63* and *Fbxo32* (Moresi et al., 2010). Together, the differential expression profile of transcriptional factors suggests that muscle atrophy in cancer cachexia can only be understood in the context of simultaneous signaling pathways activated by different transcription factors. This result is in contrast with a previous report indicating that Foxo is a single master regulator controlling muscle wasting during cancer cachexia (Judge et al., 2014). The combined action of transcriptional factors is supported by the long-standing view that specific combinations of transcriptional factors act cooperatively or in sequential steps in the regulation of gene expression (Reiter et al., 2017). Also, it has been recently demonstrated that the combination of the transcriptional factors NF-κB, SRF, and IRF controls gene expression through logical OR gates in macrophages (Cheng et al., 2017). Moreover, the cooperative interaction of the transcriptional factors STAT3 and NF-κB promotes muscle atrophy (Ma et al., 2017), further demonstrating cooperative actions of transcription factors controlling gene expression in muscle-wasting conditions.

Even though transcriptional factors are essential to understanding gene regulation, it is also crucial to identify post-transcriptional regulation mediated by miRNAs, which induce mRNA decay and inhibit translation (Bartel, 2018). To better understand the miRNA-mRNA interactome in cancer cachexia, we combined the miRNA-mRNAs expression co-profiles in the same set of muscle samples. This analysis allowed us to identify biological processes such as ECM, cell migration, and transcription as regulated by miRNAs during muscle wasting in cancer cachexia. Within these interactions, we highlight the up-regulation of miR-29b-3p, which regulates a plethora of processes such as migration, ECM, and myogenesis. The miRNA miR-29b-3p has been described as a critical regulator in a variety of processes, such as myogenesis (Wang et al., 2008) and muscle atrophy (Li et al., 2017; Moraes et al., 2017), and it has a multiplicity of targets in skeletal muscle cells, including collagen transcripts (Zhou et al., 2012b), *YY1* (Zhou et al., 2012a), *p85* (Park et al., 2009), and *IGF-1* (Gao et al., 2016).

It is worth noting that the same cancer cachexia mouse model as ours has been previously used to identify the global miRNA expression profile in wasting muscles (Lee et al., 2017), but only miR-223-3p was shared as deregulated with our data. Additionally, our set of deregulated miRNAs does not overlap with the muscle miRNA expression profile from other mice models of cancer cachexia or cachectic patients (Soares et al., 2014; Narasimhan et al., 2017). These discrepancies may be a consequence of the highly dynamic post-transcriptional regulation mechanisms exerted by miRNAs, which may act to buffer fluctuations in gene expression and confer robustness in signaling outcomes for specific regulatory networks (Ebert and Sharp, 2012). Another plausible explanation is that different miRNAs sharing the same seed regions target the same gene transcripts or pathways during the development of muscle wasting (Ebert and Sharp, 2012). Consequently, target contextual features determine miRNA target recognition and regulatory outcome as well as RNA interaction networks in specific biological contexts (Carroll et al., 2014), reinforcing the importance of the use of parallel expression profiles of mRNA and miRNA, as used herein, to gain further insights into post-transcriptional controls underlying muscle wasting in cancer cachexia.

Collectively, our integrated mRNA and miRNA data pointed out the remodeling of the ECM components in the wasting muscles. These data are in line with previous studies showing down-regulation of ECM genes in cachectic muscles of tumor-bearing mice and cancer patients (Gallagher et al., 2012; Judge et al., 2014; Moraes et al., 2017; Talbert et al., 2019). Muscle fibrosis in cachectic patients based on histological analysis has also been described in cancer cachexia (Judge et al., 2018), but these data remain to be validated at the RNA and protein levels. Therefore, determining the molecular mechanisms that lead to remodeling of the ECM and its relation to atrophy in cachexia justify further investigations.

While our study identified transcriptional and post-transcriptional regulatory networks underlying muscle wasting in cancer cachexia, our *in silico* approach is limited in several aspects. First, most studies, if not all, focusing on rodent models, suffer from limitations. Although the LLC mouse model has been extensively useful in elucidating several mechanisms of tissue wasting, it does not fully recapitulate the phenotype of human cancers, either by not forming spontaneous tumors or because of their inability to reconstitute a tumor microenvironment (Talbert et al., 2019). Second, although our work describes the variability in the expression of specific sets of genes within LLC samples, further studies are needed to establish how inter-individual variation in gene expression affects the prevalence and severity of the syndrome. Finally, our study reveals several transcription factors potentially involved in the regulation of cachexia genetic program, and experiments are still necessary to quantify their sequence-specific DNA-binding activity. In the same way, we predicted miRNA-target interactions that deserve experimental verification.

## Conclusion

In conclusion, our integrative analysis of miRNA-mRNA co-profiles comprehensively characterized regulatory relationships of molecular pathways, including miRNAs targeting ECM-associated genes, which may play a role in the development of muscle atrophy in cancer cachexia. The *in silico* analyses of the transcriptome data also revealed that these ECM genes are potentially regulated post-transcriptionally by miRNAs, such as miR-29a-3p, and transcriptionally by the NF-κB and Ciita transcription factors.

## Data Availability Statement

The datasets generated for this study can be found in the Gene Expression Omnibus (GEO) DataSets (https://www.ncbi.nlm.nih.gov/gds) under the accession numbers GSE144567 (mRNAs) and GSE145393 (miRNAs).

## Ethics Statement

The animal study was reviewed and approved by Institute of Bioscience of Botucatu Ethics Committee on Animal Use, from the São Paulo State University (UNESP, Brazil).

## Author Contributions

GF, LM, PF, JG, MD-P-S, and RC: conceptualization. GF, JF, IV-J, LM, SC, PF, JG, and RF: data curation. GF, IV-J, SC, PF, and JG: formal analysis. GF, MD-P-S, and RC: funding acquisition. GF, JF, IV-J, LM, SC, PF, RF, SR, and RC: investigation. GF and RF: methodology. GF, MD-P-S, and RC: project administration. RC: resources and supervision. GF: software, visualization, and writing – original draft. All authors performend writing – review, editing, read the manuscript, and have agreed to be co-authors.

## Conflict of Interest

The authors declare that the research was conducted in the absence of any commercial or financial relationships that could be construed as a potential conflict of interest.

## Footnotes

1 http://159.149.160.88/pscan/

2 http://string-db.org/

3 https://cytoscape.org/

## Supplementary Material

FIGURE S1 **(a)** The exposed carcass of control and Lewis Lung Cancer (LLC) tumor-bearing mice, twenty-two days after subcutaneous injection of PBS or 1.5 × 10^6^ LLC cells, respectively. **(b)** Tumor mass of LLC tumor-bearing mice. **(c)** Splenomegaly in LCC tumor-bearing mouse compared to a control.

FIGURE S2 mRNA levels of the genes involved in myogenesis, sarcomere, proteasome, and ECM in LLC and control groups. RT-qPCR data are presented as Log2 fold change (2^−ΔΔ*Ct*^) relative to Rpl13a. Statistical difference was analyzed by Student’s *t*-test. ^∗^*P* < 0.05.

FIGURE S3 **(a)** Gene-Ontology analysis of differentially expressed genes (DEG) from control vs. tumor-bearing mice (LLC) for individual clusters (I to IV) identified by the unsupervised hierarchical clustering analysis (shown in Figure 2B). Each horizontal gray bar represents the gene fold enrichment compared to the total number of genes in each ontology term. **(b)** Hierarchical clustering of the Pearson correlation values identified LLC subgroups represented by colored lines in the dendrogram: control group (light blue; C1–C4), LLC group (pink; L1–L6), subgroup A (red; L2–L4), and subgroup B (black; L5 and L6). **(c)** Bar plot representing the total number of up- and down-regulated genes (red and blue, respectively) in each subgroup of samples identified in LLC (as shown in B). **(d)** Heatmap of 443 Z-score normalized DEG identified in LLC subgroup A (L2–L4) vs. control (C1–C4) analyzed by unsupervised hierarchical clustering. Down- and up-regulated genes with absolute values of fold-change > 1.5 and FDR < 0.05 (Wald test) are shown in red and blue, respectively. Subgroup A-cumulative frequency distribution of the DEG (log2-fold change, x-axis) from LLC (L1–L6) vs. control samples, indicated as a percentage (%, y-axis) for up- and down-regulated genes in **(e)** and **(f)**, respectively. **(g)** Gene-Ontology analysis of DEG from control vs. LLC samples from subgroup A. Each horizontal black bar represents the gene fold enrichment compared to the total number of genes in each ontology term.

FIGURE S4 mRNA levels of the genes involved with ECM in different muscle types from LLC and control groups. RT-qPCR data are presented as Log2 fold change (2^−ΔΔ*Ct*^) relative to Rpl13a. Statistical difference was analyzed by Student’s *t*-test. ^∗^*P* < 0.05.

TABLE S1 Anatomical Data of control and tumor-bearing mice (LLC) groups.

TABLE S2 Differentially expressed genes in Tibialis Anterior muscle in LLC tumor-bearing mice.

TABLE S3 Functional classification of differentially expressed genes in Tibialis Anterior muscle in LLC tumor-bearing mice.

TABLE S4 Differentially expressed miRNAs in Tibialis Anterior muscle in LLC tumor-bearing mice.

## References

[B1] Agarwal P. Srivastava R. Srivastava A. K. Ali S. Datta M. (2013). MiR-135a targets IRS2 and regulates insulin signaling and glucose uptake in the diabetic gastrocnemius skeletal muscle. *Biochim. Biophys. Acta Mol. Basis Dis.* 1832 1294–1303. 10.1016/j.bbadis.2013.03.021 23579070

[B2] An H. T. Kim J. Yoo S. Ko J. (2014). Small leucine zipper protein (sLZIP) negatively regulates skeletal muscle differentiation via interaction withɑ-actinin-4. *J. Biol. Chem.* 289 4969–4979. 10.1074/jbc.M113.515395 24375477 PMC3931057

[B3] Anders S. Pyl P. T. Huber W. (2015). HTSeq-A Python framework to work with high-throughput sequencing data. *Bioinformatics* 31 166–169. 10.1093/bioinformatics/btu638 25260700 PMC4287950

[B4] Asp P. Acosta-Alvear D. Tsikitis M. van Oevelen C. Dynlacht B. D. (2009). E2f3b plays an essential role in myogenic differentiation through isoform-specific gene regulation. *Genes Dev.* 23 37–53. 10.1101/gad.1727309 19136625 PMC2632163

[B5] Baracos V. E. Martin L. Korc M. Guttridge D. C. Fearon K. C. H. (2018). Cancer-associated cachexia. *Nat. Rev. Dis. Prim.* 4 1–18. 10.1038/nrdp.2017.105 29345251

[B6] Bartel D. P. (2018). Metazoan microRNAs. *Cell* 173 20–51. 10.1016/j.cell.2018.03.006 29570994 PMC6091663

[B7] Benjamini Y. Hochberg Y. (1995). Controlling the false discovery rate: a practical and powerful approach to multiple testing. *J. R. Stat. Soc. Ser. B* 57 289–300. 10.1111/j.2517-6161.1995.tb02031.x

[B8] Berkes C. A. Bergstrom D. A. Penn B. H. Seaver K. J. Knoepfler P. S. Tapscott S. J. (2004). Pbx marks genes for activation by MyoD indicating a role for a homeodomain protein in establishing myogenic potential. *Mol. Cell* 14 465–477. 10.1016/s1097-2765(04)00260-6 15149596

[B9] Bindea G. Mlecnik B. Hackl H. Charoentong P. Tosolini M. Kirilovsky A. (2009). ClueGO: a Cytoscape plug-in to decipher functionally grouped gene ontology and pathway annotation networks. *Bioinformatics* 25 1091–1093. 10.1093/bioinformatics/btp101 19237447 PMC2666812

[B10] Bodine S. C. Latres E. Baumhueter S. Lai V. K. Nunez L. Clarke B. A. (2001). Identification of ubiquitin ligases required for skeletal muscle atrophy. *Science* 294 1704–1708. 10.1126/science.1065874 11679633

[B11] Bonetto A. Aydogdu T. Kunzevitzky N. Guttridge D. C. Khuri S. Koniaris L. G. (2011). STAT3 activation in skeletal muscle links muscle wasting and the acute phase response in cancer cachexia. *PLoS One* 6:e22538. 10.1371/journal.pone.0022538 21799891 PMC3140523

[B12] Bradford M. M. (1976). A rapid and sensitive method for the quantitation of microgram quantities of protein utilizing the principle of protein-dye binding. *Anal. Biochem.* 72 248–254. 10.1006/abio.1976.9999 942051

[B13] Cai D. Frantz J. D. Tawa N. E. Melendez P. A. Oh B.-C. Lidov H. G. W. (2004). IKKbeta/NF-kappaB activation causes severe muscle wasting in mice. *Cell* 119 285–298. 10.1016/j.cell.2004.09.027 15479644

[B14] Carroll A. P. Goodall G. J. Liu B. (2014). Understanding principles of miRNA target recognition and function through integrated biological and bioinformatics approaches. *Wiley Interdiscip. Rev. RNA* 5 361–379. 10.1002/wrna.1217 24459110

[B15] Chen J. L. Walton K. L. Hagg A. Colgan T. D. Johnson K. Qian H. (2017). Specific targeting of TGF-β family ligands demonstrates distinct roles in the regulation of muscle mass in health and disease. *Proc. Natl. Acad. Sci. U.S.A.* 114 E5266–E5275. 10.1073/pnas.1620013114 28607086 PMC5495232

[B16] Chen W. Zhang X. Birsoy K. Roeder R. G. (2010). A muscle-specific knockout implicates nuclear receptor coactivator MED1 in the regulation of glucose and energy metabolism. *Proc. Natl. Acad. Sci. U.S.A.* 107 10196–10201. 10.1073/pnas.1005626107 20479251 PMC2890439

[B17] Cheng C. S. Behar M. S. Suryawanshi G. W. Feldman K. E. Spreafico R. Hoffmann A. (2017). Iterative modeling reveals evidence of sequential transcriptional control mechanisms. *Cell Syst.* 4 330–343.e5. 10.1016/j.cels.2017.01.012 28237795 PMC5434763

[B18] Choi M.-C. Cohen T. J. Barrientos T. Wang B. Li M. Simmons B. J. (2012). A direct HDAC4-MAP kinase crosstalk activates muscle atrophy program. *Mol. Cell* 47 122–132. 10.1016/j.molcel.2012.04.025 22658415 PMC3398192

[B19] Costelli P. Muscaritoli M. Bossola M. Crepaldi S. Sperimentale O. Torino U. (2005). Skeletal muscle wasting in tumor-bearing rats is associated with MyoD down-regulation. *Int. J. Oncol.* 26 1663–1668. 10.3892/ijo.26.6.1663 15870883

[B20] Dewys W. D. Begg C. Lavin P. T. Band P. R. Bennett J. M. Bertino J. R. (1980). Prognostic effect of weight loss prior to chemotherapy in cancer patients. Eastern Cooperative Oncology Group. . *Am. J. Med.* 69 491–497. 10.1016/s0149-2918(05)80001-3 7424938

[B21] Dhanapal R. Saraswathi T. Govind R. N. (2011). Cancer cachexia. *J. Oral Maxillofac. Pathol.* 15 257–260. 10.4103/0973-029X.86670 22144825 PMC3227249

[B22] Dragomir M. P. Knutsen E. Calin G. A. (2018). SnapShot: unconventional miRNA functions. *Cell* 174 1038–1038.e1. 10.1016/j.cell.2018.07.040 30096304

[B23] Ebert M. S. Sharp P. A. (2012). Roles for microRNAs in conferring robustness to biological processes. *Cell* 149 515–524. 10.1016/j.cell.2012.04.005 22541426 PMC3351105

[B24] Ecker S. Pancaldi V. Rico D. Valencia A. (2015). Higher gene expression variability in the more aggressive subtype of chronic lymphocytic leukemia. *Genome Med.* 7:8. 10.1186/s13073-014-0125-z 25632304 PMC4308895

[B25] Eisenberg I. Eran A. Nishino I. Moggio M. Lamperti C. Amato A. A. (2007). Distinctive patterns of microRNA expression in primary muscular disorders. *Proc. Natl. Acad. Sci. U.S.A.* 104 17016–17021. 10.1073/pnas.0708115104 17942673 PMC2040449

[B26] Eldar A. Elowitz M. B. (2010). Functional roles for noise in genetic circuits. *Nature* 467 167–173. 10.1038/nature09326 20829787 PMC4100692

[B27] Fearon K. Arends J. Baracos V. (2013). Understanding the mechanisms and treatment options in cancer cachexia. *Nat. Rev. Clin. Oncol.* 10 90–99. 10.1038/nrclinonc.2012.209 23207794

[B28] Fearon K. C. H. Glass D. J. Guttridge D. C. (2012). Cancer cachexia: mediators, signaling, and metabolic pathways. *Cell Metab.* 16 153–166. 10.1016/j.cmet.2012.06.011 22795476

[B29] Fernandez G. J. Ferreira J. H. Vechetti-Júnior I. J. de Moraes L. N. Cury S. S. Freire P. P. (2019). MicroRNA-mRNA co-sequencing identifies transcriptional and post-transcriptional regulatory networks underlying muscle wasting in cancer cachexia. [Preprints]. 10.20944/preprints201909.0004.v1 PMC7272700 32547603

[B30] Flynt A. S. Lai E. C. (2008). Biological principles of microRNA-mediated regulation: shared themes amid diversity. *Nat. Rev. Genet.* 9 831–842. 10.1038/nrg2455 18852696 PMC2729318

[B31] Fontes-Oliveira C. C. Busquets S. Fuster G. Ametller E. Figueras M. Olivan M. (2014). A differential pattern of gene expression in skeletal muscle of tumor-bearing rats reveals dysregulation of excitation–contraction coupling together with additional muscle alterations. *Muscle Nerve* 49 233–248. 10.1002/mus.23893 23649607

[B32] Fukawa T. Yan-Jiang B. C. Min-Wen J. C. Jun-Hao E. T. Huang D. Qian C.-N. (2016). Excessive fatty acid oxidation induces muscle atrophy in cancer cachexia. *Nat. Med.* 22 666–671. 10.1038/nm.4093 27135739

[B33] Gallagher I. J. Stephens N. A. MacDonald A. J. Skipworth R. J. E. Husi H. Greig C. A. (2012). Suppression of skeletal muscle turnover in cancer cachexia: evidence from the transcriptome in sequential human muscle biopsies. *Clin. Cancer Res.* 18 2817–2827. 10.1158/1078-0432.CCR-11-2133 22452944

[B34] Gao S. Cheng C. Chen H. Li M. Liu K. Wang G. (2016). IGF1 3’UTR functions as a ceRNA in promoting angiogenesis by sponging miR-29 family in osteosarcoma. *J. Mol. Histol.* 47 135–143. 10.1007/s10735-016-9659-2 26759259

[B35] Garcia D. M. Baek D. Shin C. Bell G. W. Grimson A. Bartel D. P. (2011). Weak seed-pairing stability and high target-site abundance decrease the proficiency of lsy-6 and other microRNAs. *Nat. Struct. Mol. Biol.* 18 1139–1146. 10.1038/nsmb.2115 21909094 PMC3190056

[B36] Gerstein M. B. Kundaje A. Hariharan M. Landt S. G. Yan K.-K. Cheng C. (2012). Architecture of the human regulatory network derived from ENCODE data. *Nature* 489 91–100. 10.1038/nature11245 22955619 PMC4154057

[B37] Grifone R. Laclef C. Spitz F. Lopez S. Demignon J. Guidotti J.-E. (2004). Six1 and Eya1 expression can reprogram adult muscle from the slow-twitch phenotype into the fast-twitch phenotype. *Mol. Cell. Biol.* 24 6253–6267. 10.1128/MCB.24.14.6253-6267.2004 15226428 PMC434262

[B38] Guan J. Yang E. Yang J. Zeng Y. Ji G. Cai J. J. (2016). Exploiting aberrant mRNA expression in autism for gene discovery and diagnosis. *Hum. Genet.* 135 797–811. 10.1007/s00439-016-1673-7 27131873

[B39] Hsu S.-D. Lin F.-M. Wu W.-Y. Liang C. Huang W.-C. Chan W.-L. (2011). miRTarBase: a database curates experimentally validated microRNA-target interactions. *Nucleic Acids Res.* 39 D163–D169. 10.1093/nar/gkq1107 21071411 PMC3013699

[B40] Jheng J. R. Chen Y. S. Ao U. I. Chan D. C. Huang J. W. Hung K. Y. (2018). The double-edged sword of endoplasmic reticulum stress in uremic sarcopenia through myogenesis perturbation. *J. Cachexia Sarcopenia Muscle* 9 570–584. 10.1002/jcsm.12288 29380555 PMC5989876

[B41] Johns N. Stretch C. Tan B. H. L. Solheim T. S. Sørhaug S. Stephens N. A. (2017). New genetic signatures associated with cancer cachexia as defined by low skeletal muscle index and weight loss. *J. Cachexia Sarcopenia Muscle* 8 122–130. 10.1002/jcsm.12138 27897403 PMC5356227

[B42] Judge S. M. Nosacka R. L. Delitto D. Gerber M. H. Cameron M. E. Trevino J. G. (2018). Skeletal muscle fibrosis in pancreatic cancer patients with respect to survival. *JNCI Cancer Spectr.* 2:ky043. 10.1093/jncics/pky043 30637373 PMC6322478

[B43] Judge S. M. Wu C.-L. Beharry A. W. Roberts B. M. Ferreira L. F. Kandarian S. C. (2014). Genome-wide identification of FoxO-dependent gene networks in skeletal muscle during C26 cancer cachexia. *BMC Cancer* 14:997. 10.1186/1471-2407-14-997 25539728 PMC4391468

[B44] Kim D. Pertea G. Trapnell C. Pimentel H. Kelley R. Salzberg S. L. (2013). TopHat2: accurate alignment of transcriptomes in the presence of insertions, deletions and gene fusions. *Genome Biol.* 14:R36. 10.1186/gb-2013-14-4-r36 23618408 PMC4053844

[B45] Kirby L. S. Kirby M. A. Warren J. W. Tran L. T. Yellon S. M. (2005). Increased innervation and ripening of the prepartum murine cervix. *J. Soc. Gynecol. Investig.* 12 578–585. 10.1016/j.jsgi.2005.08.006 16325747

[B46] Krek A. Grün D. Poy M. N. Wolf R. Rosenberg L. Epstein E. J. (2005). Combinatorial microRNA target predictions. *Nat. Genet.* 37 495–500. 10.1038/ng1536 15806104

[B47] LaBarge S. McDonald M. Smith-Powell L. Auwerx J. Huss J. M. (2014). Estrogen-related receptor-α (ERRα) deficiency in skeletal muscle impairs regeneration in response to injury. *FASEB J.* 28 1082–1097. 10.1096/fj.13-229211 24277576 PMC3929682

[B48] Langmead B. Trapnell C. Pop M. Salzberg S. (2009). Ultrafast and memory-efficient alignment of short DNA sequences to the human genome. *Genome Biol.* 10:R25. 10.1186/gb-2009-10-3-r25 19261174 PMC2690996

[B49] Lee D. E. Brown J. L. Rosa-Caldwell M. E. Blackwell T. A. Perry R. A. Brown L. A. (2017). Cancer cachexia-induced muscle atrophy: evidence for alterations in microRNAs important for muscle size. *Physiol. Genomics* 49 253–260. 10.1152/physiolgenomics.00006.2017 28341621

[B50] Li J. Chan M. C. Yu Y. Bei Y. Chen P. Zhou Q. (2017). MiR-29b contributes to multiple types of muscle atrophy. *Nat. Commun.* 8:15201. 10.1038/ncomms15201 28541289 PMC5458521

[B51] Loberg R. D. Bradley D. A. Tomlins S. A. Chinnaiyan A. M. Pienta K. J. (2007). The lethal phenotype of cancer: the molecular basis of death due to malignancy. *CA Cancer J. Clin.* 57 225–241. 10.3322/canjclin.57.4.225 17626119

[B52] Love M. I. Huber W. Anders S. (2014). Moderated estimation of fold change and dispersion for RNA-seq data with DESeq2. *Genome Biol.* 15:550. 10.1186/s13059-014-0550-8 25516281 PMC4302049

[B53] Ma J. F. Sanchez B. J. Hall D. T. Tremblay A. K. Di Marco S. Gallouzi I. (2017). STAT3 promotes IFNγ/TNFα-induced muscle wasting in an NF-κB-dependent and IL-6-independent manner. *EMBO Mol. Med.* 9 622–637. 10.15252/emmm.201607052 28264935 PMC5412921

[B54] Mar J. C. Matigian N. A. Mackay-Sim A. Mellick G. D. Sue C. M. Silburn P. A. (2011). Variance of gene expression identifies altered network constraints in neurological disease. *PLoS Genet.* 7:1002207. 10.1371/journal.pgen.1002207 21852951 PMC3154954

[B55] Maragkakis M. Reczko M. Simossis V. A. Alexiou P. Papadopoulos G. L. Dalamagas T. (2009). DIANA-microT web server: elucidating microRNA functions through target prediction. *Nucleic Acids Res.* 37 W273–W276. 10.1093/nar/gkp292 19406924 PMC2703977

[B56] Martin L. Birdsell L. MacDonald N. Reiman T. Clandinin M. T. McCargar L. J. (2013). Cancer cachexia in the age of obesity: skeletal muscle depletion is a powerful prognostic factor, independent of body mass index. *J. Clin. Oncol.* 31 1539–1547. 10.1200/JCO.2012.45.2722 23530101

[B57] Matsuyama T. Ishikawa T. Okayama T. Oka K. Adachi S. Mizushima K. (2015). Tumor inoculation site affects the development of cancer cachexia and muscle wasting. *Int. J. Cancer* 137 2558–2565. 10.1002/ijc.29620 26016447

[B58] Monitto C. L. Berkowitz D. Lee K. M. Pin S. Li D. Breslow M. (2001). Differential gene expression in a murine model of cancer cachexia. *Am. J. Physiol. Endocrinol. Metab.* 281 E289–E297. 10.1152/ajpendo.2001.281.2.E289 11440905

[B59] Moraes L. N. Fernandez G. J. Vechetti-Júnior I. J. Freire P. P. Souza R. W. A. Villacis R. A. R. (2017). Integration of miRNA and mRNA expression profiles reveals microRNA-regulated networks during muscle wasting in cardiac cachexia. *Sci. Rep.* 7:6998. 10.1038/s41598-017-07236-2 28765595 PMC5539204

[B60] Moresi V. Williams A. H. Meadows E. Flynn J. M. Potthoff M. J. McAnally J. (2010). Myogenin and class II HDACs control neurogenic muscle atrophy by inducing E3 ubiquitin ligases. *Cell* 143 35–45. 10.1016/j.cell.2010.09.004 20887891 PMC2982779

[B61] Moretti I. Ciciliot S. Dyar K. A. Abraham R. Murgia M. Agatea L. (2016). MRF4 negatively regulates adult skeletal muscle growth by repressing MEF2 activity. *Nat. Commun.* 7:12397. 10.1038/ncomms12397 27484840 PMC4976255

[B62] Naguibneva I. Ameyar-Zazoua M. Polesskaya A. Ait-Si-Ali S. Groisman R. Souidi M. (2006). The microRNA miR-181 targets the homeobox protein Hox-A11 during mammalian myoblast differentiation. *Nat. Cell Biol.* 8 278–284. 10.1038/ncb1373 16489342

[B63] Narasimhan A. Ghosh S. Stretch C. Greiner R. Bathe O. F. Baracos V. (2017). Small RNAome profiling from human skeletal muscle: novel miRNAs and their targets associated with cancer cachexia. *J. Cachexia Sarcopenia Muscle* 8 405–416. 10.1002/jcsm.12168 28058815 PMC5476855

[B64] Norden D. M. Devine R. McCarthy D. O. Wold L. E. (2015). Storage Conditions and Passages Alter IL-6 secretion in C26 adenocarcinoma cell lines. *MethodsX* 2 53–58. 10.1016/j.mex.2015.02.001 25709898 PMC4335308

[B65] Nuoc T.-N. Kim S. Ahn S. H. Lee J.-S. Park B.-J. Lee T.-H. (2017). The analysis of antioxidant expression during muscle atrophy induced by hindlimb suspension in mice. *J. Physiol. Sci.* 67 121–129. 10.1007/s12576-016-0444-5 26971264 PMC10717164

[B66] Pacagnelli F. L. Sabela A. K. Mariano T. B. Ozaki G. A. T. Castoldi R. C. Carmo E. M. (2016). Fractal dimension in quantifying experimental-pulmonary-hypertension-induced cardiac dysfunction in rats. *Arq. Bras. Cardiol.* 107 33–39. 10.5935/abc.20160083 27223643 PMC4976954

[B67] Pacini L. Suffredini S. Ponti D. Coppini R. Frati G. Ragona G. (2013). Altered calcium regulation in isolated cardiomyocytes from *Egr-1* knock-out mice. *Can. J. Physiol. Pharmacol.* 91 1135–1142. 10.1139/cjpp-2012-0419 24289086

[B68] Park S. Y. Lee J. H. Ha M. Nam J. W. Kim V. N. (2009). miR-29 miRNAs activate p53 by targeting p85α and CDC42. *Nat. Struct. Mol. Biol.* 16 23–29. 10.1002/jqs.1537 19079265

[B69] Porporato P. E. (2016). Understanding cachexia as a cancer metabolism syndrome. *Oncogenesis* 5 e200–e210. 10.1038/oncsis.2016.3 26900952 PMC5154342

[B70] Prado C. M. Sawyer M. B. Ghosh S. Lieffers J. R. Esfandiari N. Antoun S. (2013). Central tenet of cancer cachexia therapy: do patients with advanced cancer have exploitable anabolic potential? *Am. J. Clin. Nutr.* 98 1012–1019. 10.3945/ajcn.113.060228 23966429

[B71] Reiter F. Wienerroither S. Stark A. (2017). Combinatorial function of transcription factors and cofactors. *Curr. Opin. Genet. Dev.* 43 73–81. 10.1016/j.gde.2016.12.007 28110180

[B72] Rossi G. Antonini S. Bonfanti C. Monteverde S. Vezzali C. Tajbakhsh S. (2016). Nfix regulates temporal progression of muscle regeneration through modulation of myostatin expression. *Cell Rep.* 14 2238–2249. 10.1016/j.celrep.2016.02.014 26923583 PMC4793149

[B73] Shannon P. Markiel A. Ozier O. Baliga N. S. Wang J. T. Ramage D. (2003). Cytoscape: a software environment for integrated models of biomolecular interaction networks. *Genome Res.* 13 2498–2504. 10.1101/gr.1239303 14597658 PMC403769

[B74] Shen H. Liu T. Fu L. Zhao S. Fan B. Cao J. (2013). Identification of microRNAs involved in dexamethasone-induced muscle atrophy. *Mol. Cell. Biochem.* 381 105–113. 10.1007/s11010-013-1692-9 23716137

[B75] Shum A. M. Y. Fung D. C. Y. Corley S. M. McGill M. C. Bentley N. Tan T. C. (2015). Cardiac and skeletal muscles show molecularly distinct responses to cancer cachexia. *Physiol. Genomics* 47 588–599. 10.1152/physiolgenomics.00128.2014 26395599

[B76] Snel B. Lehmann G. Bork P. Huynen M. A. (2000). STRING: a web-server to retrieve and display the repeatedly occurring neighbourhood of a gene. *Nucleic Acids Res.* 28 3442–3444. 10.1093/nar/28.18.3442 10982861 PMC110752

[B77] Soares R. J. Cagnin S. Chemello F. Silvestrin M. Musaro A. De Pitta C. (2014). Involvement of microRNAs in the regulation of muscle wasting during catabolic conditions. *J. Biol. Chem.* 289 21909–21925. 10.1074/jbc.M114.561845 24891504 PMC4139209

[B78] Stephens N. A. Gallagher I. J. Rooyackers O. Skipworth R. J. Tan B. H. Marstrand T. (2010). Using transcriptomics to identify and validate novel biomarkers of human skeletal muscle cancer cachexia. *Genome Med.* 2:1. 10.1186/gm122 20193046 PMC2829926

[B79] Summermatter S. Bouzan A. Pierrel E. Melly S. Stauffer D. Gutzwiller S. (2017). Blockade of Metallothioneins 1 and 2 increases skeletal muscle mass and strength. *Mol. Cell. Biol.* 37 e305–e316. 10.1128/MCB.00305-16 27956698 PMC5311239

[B80] Sun Y. Li Y. Wang H. Li H. Liu S. Chen J. (2017). miR-146a-5p acts as a negative regulator of TGF-β signaling in skeletal muscle after acute contusion. *Acta Biochim. Biophys. Sin.* 49 628–634. 10.1093/abbs/gmx052 28510617

[B81] Szklarczyk D. Morris J. H. Cook H. Kuhn M. Wyder S. Simonovic M. (2017). The STRING database in 2017: quality-controlled protein–protein association networks, made broadly accessible. *Nucleic Acids Res.* 45 D362–D368. 10.1093/nar/gkw937 27924014 PMC5210637

[B82] Talbert E. E. Cuitiño M. C. Ladner K. J. Rajasekerea P. V. Siebert M. Shakya R. (2019). Modeling human cancer-induced cachexia. *Cell Rep* 28 1612–1622.e4. 10.1016/j.celrep.2019.07.016 31390573 PMC6733019

[B83] Tsika R. W. Schramm C. Simmer G. Fitzsimons D. P. Moss R. L. Ji J. (2008). Overexpression of TEAD-1 in transgenic mouse striated muscles produces a slower skeletal muscle contractile phenotype. *J. Biol. Chem.* 283 36154–36167. 10.1074/jbc.M807461200 18978355 PMC2606011

[B84] Vaughan V. C. Martin P. Lewandowski P. A. (2013). Cancer cachexia: impact, mechanisms and emerging treatments. *J. Cachexia Sarcopenia Muscle* 4 95–109. 10.1007/s13539-012-0087-1 23097000 PMC3684701

[B85] von Haehling S. Anker M. S. Anker S. D. (2016). Prevalence and clinical impact of cachexia in chronic illness in Europe, USA, and Japan: facts and numbers update 2016. *J. Cachexia Sarcopenia Muscle* 7 507–509. 10.1002/jcsm.12167 27891294 PMC5114624

[B86] Wang H. Garzon R. Sun H. Ladner K. J. Singh R. Dahlman J. (2008). NF-kappaB-YY1-miR-29 regulatory circuitry in skeletal myogenesis and rhabdomyosarcoma. *Cancer Cell* 14 369–381. 10.1016/j.ccr.2008.10.006 18977326 PMC3829205

[B87] Wang X. (2008). miRDB: a microRNA target prediction and functional annotation database with a wiki interface. *RNA* 14 1012–1017. 10.1261/rna.965408 18426918 PMC2390791

[B88] Watanabe S. Kondo S. Hayasaka M. Hanaoka K. (2007). Functional analysis of homeodomain-containing transcription factor Lbx1 in satellite cells of mouse skeletal muscle. *J. Cell Sci.* 120 4178–4187. 10.1242/jcs.011668 18003701

[B89] Zambelli F. Pesole G. Pavesi G. (2009). Pscan: finding over-represented transcription factor binding site motifs in sequences from co-regulated or co-expressed genes. *Nucleic Acids Res.* 37 W247–W252. 10.1093/nar/gkp464 19487240 PMC2703934

[B90] Zhang F. Shugart Y. Y. Yue W. Cheng Z. Wang G. Zhou Z. (2015). Increased variability of genomic transcription in schizophrenia. *Sci. Rep.* 5:17995 10.1038/srep17995 PMC4675071 26657146

[B91] Zhang L. Pan J. Dong Y. Tweardy D. J. Dong Y. Garibotto G. (2013). Stat3 activation links a C/EBPδ to myostatin pathway to stimulate loss of muscle mass. *Cell Metab.* 18 368–379. 10.1016/j.cmet.2013.07.012 24011072 PMC3794464

[B92] Zhou L. Sun K. Zhao Y. Zhang S. Wang X. Li Y. (2015). Linc-YY1 promotes myogenic differentiation and muscle regeneration through an interaction with the transcription factor YY1. *Nat. Commun.* 6:10026. 10.1038/ncomms10026 26658965

[B93] Zhou L. Wang L. Lu L. Jiang P. Sun H. Wang H. (2012a). A novel target of microRNA-29, Ring1 and YY1-binding protein (Rybp), negatively regulates skeletal myogenesis. *J. Biol. Chem.* 287 25255–25265. 10.1074/jbc.M112.357053 22661705 PMC3408151

[B94] Zhou L. Wang L. Lu L. Jiang P. Sun H. Wang H. (2012b). Inhibition of miR-29 by TGF-beta-Smad3 signaling through dual mechanisms promotes transdifferentiation of mouse myoblasts into myofibroblasts. *PLoS One* 7:e33766. 10.1371/journal.pone.0033766 22438993 PMC3306299

