# Supplementary material for: MicroRNA-mRNA Co-sequencing Identifies Transcriptional and Post-transcriptional Regulatory Networks Underlying Muscle Wasting in Cancer Cachexia
Source: Front Genet. 2020 May 29;11:541. doi: 10.3389/fgene.2020.00541 (PMC7272700; doi:10.3389/fgene.2020.00541)
Supplement: FIGURE S1 — (a) The exposed carcass of control and Lewis Lung Cancer (LLC) tumor-bearing mice, twenty-two days after subcutaneous injection of PBS or 1.5 × 10^6^ LLC cells, respectively. (b) Tumor mass of LLC tumor-bearing mice. (c) Splenomegaly in LCC tumor-bearing mouse compared to a control. [file Presentation_1.PDF]

Supplementary Files

Table S1

Anatomical variable	Control (n=6)	LLC (n=13)
Initial body weight (g)	28.5±2.3	28.7±2.6
Final body weight (g)	30.4±2.5	31.1±2.6
Tumor weight (g)	0	4.4±1.0
Final Tumor free weight (g)	ND	26.74±2.5
Heart weight (g)	0.167±0.012	0.161±0.013
Liver weight (mg)	1.622±0.23	1.684±0.21
Gastro weight (mg)	163±9	132.8±10*
Soleus weight (mg)	12±1	8.3±3*
TA weight (mg)	5.4±0.6	4.26±0.6*
Spleen weight (mg)	85±7	304±60*
Epididymal fat	424±120	295±90*
Retroperitoneal fat	155±80	56±20*
Visceral fat	608±136	449±82*

Values are mean ± SD; n: number of animals. control group (n = 6); LLC: Tumor bearing mice group (n = 13); *= P value< 0.05

Figure S1

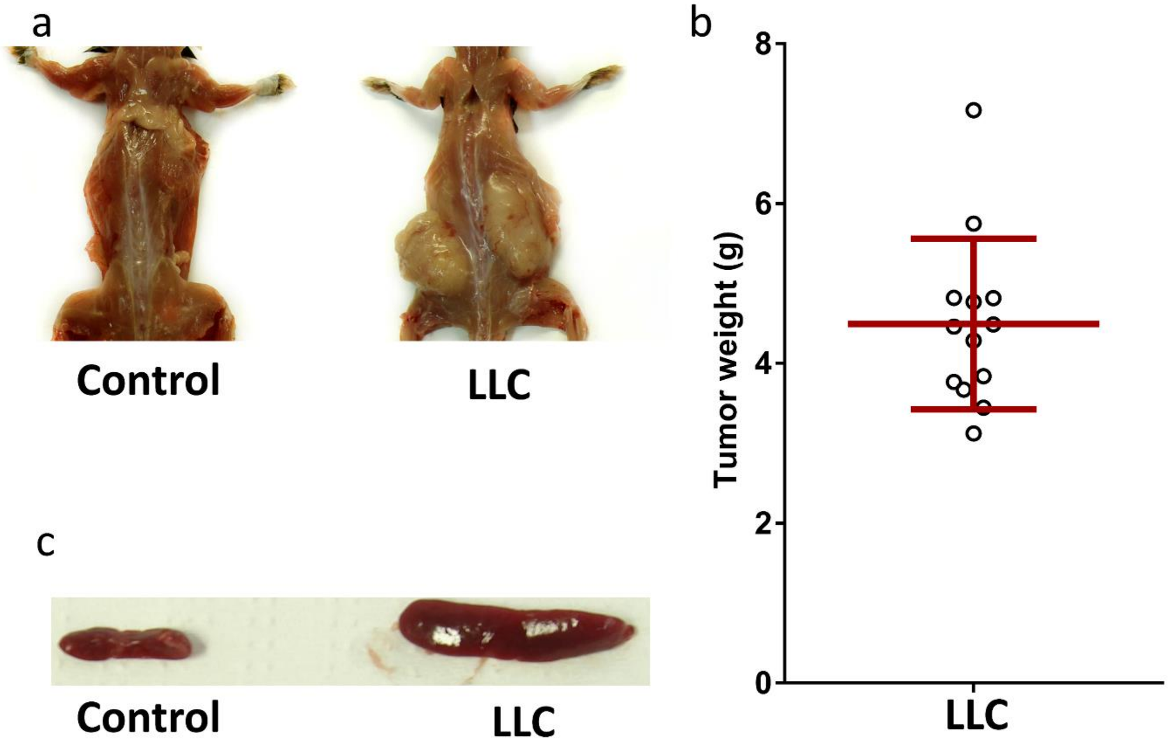

Figure S1. (a) The exposed carcass of control and Lewis Lung Cancer (LLC) tumor-bearing mice, twenty-two days after subcutaneous injection of PBS or 1.5x10^6 LLC cells, respectively. **(b)** Tumor mass of LLC tumor-bearing mice. **(c)** Splenomegaly in LCC tumor-bearing mouse compared to a control.

Figure S2

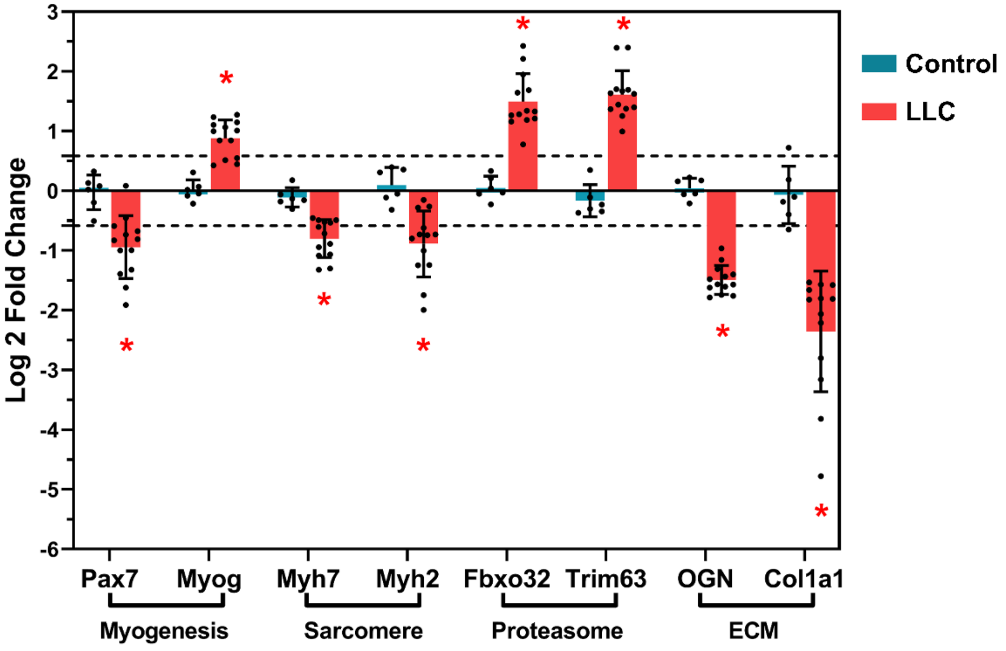

Figure S2. mRNA levels of the genes involved in myogenesis, sarcomere, proteasome, and ECM in LLC and control groups. RT-qPCR data are presented as Log2 fold change (2^-ΔΔCt) relative to Rpl13a. Statistical difference was analyzed by Student's t-test. * $P < 0.05$.

Figure S3

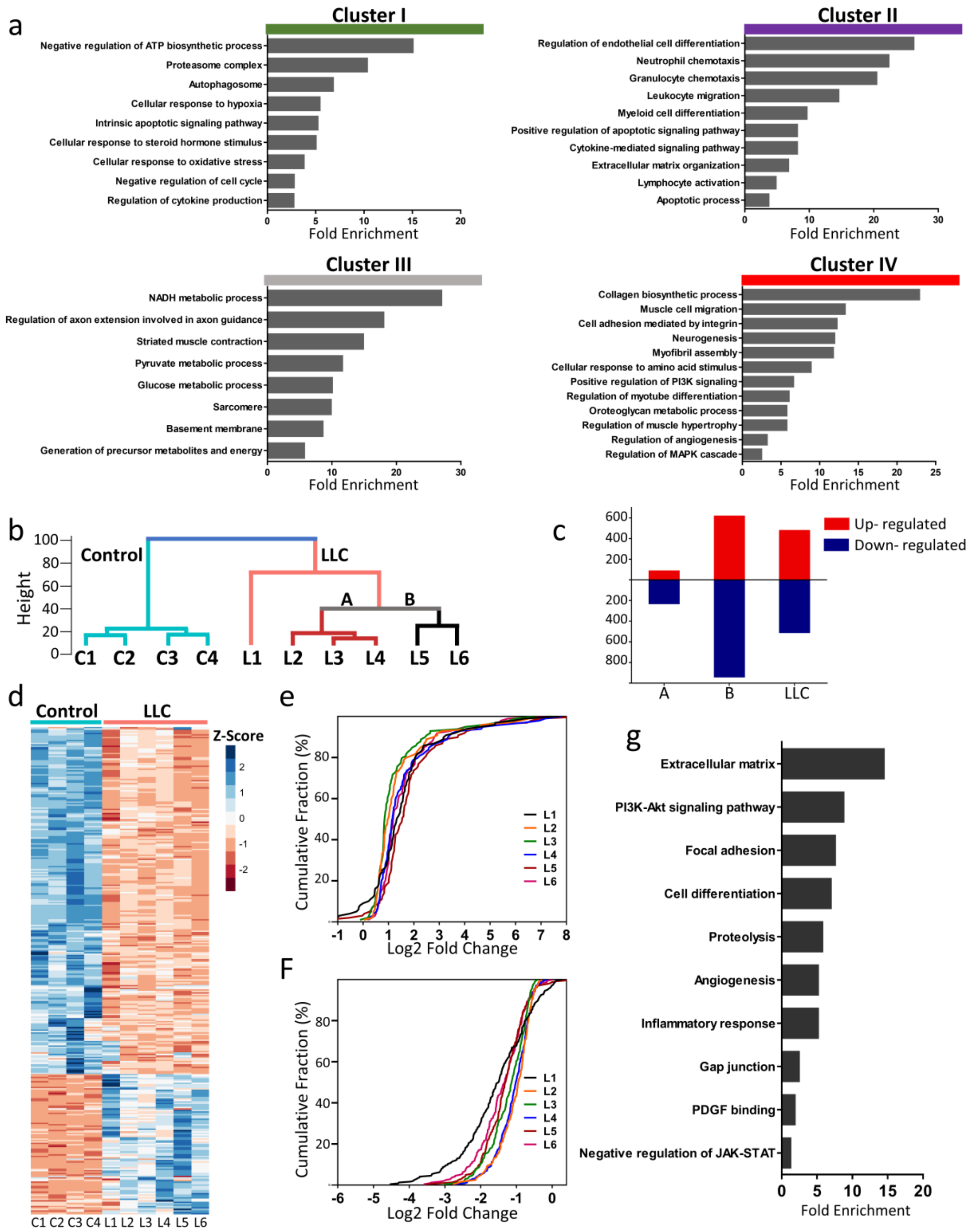
a
Cluster I
Negative regulation of ATP biosynthetic process
Proteasome complex
Autophagosome
Cellular response to hypoxia
Intrinsic apoptotic signaling pathway
Cellular response to steroid hormone stimulus
Cellular response to oxidative stress
Negative regulation of cell cycle
Regulation of cytokine production
0 5 10 15 20
Fold Enrichment
Cluster II
Regulation of endothelial cell differentiation
Neutrophil chemotaxis
Granulocyte chemotaxis
Leukocyte migration
Myeloid cell differentiation
Positive regulation of apoptotic signaling pathway
Cytokine-mediated signaling pathway
Extracellular matrix organization
Lymphocyte activation
Apoptotic process
0 10 20 30
Fold Enrichment
Cluster III
NADH metabolic process
Regulation of axon extension involved in axon guidance
Striated muscle contraction
Pyruvate metabolic process
Glucose metabolic process
Sarcomere
Basement membrane
Generation of precursor metabolites and energy
0 10 20 30
Fold Enrichment
Cluster IV
Collagen biosynthetic process
Muscle cell migration
Cell adhesion mediated by integrin
Neurogenesis
Myofibril assembly
Cellular response to amino acid stimulus
Positive regulation of PI3K signaling
Regulation of myotube differentiation
Oroteoglycan metabolic process
Regulation of muscle hypertrophy
Regulation of angiogenesis
Regulation of MAPK cascade
0 5 10 15 20 25
Fold Enrichment
b
Height
100 80 60 40 20 0
Control
LLC
A
B
C1 C2 C3 C4 L1 L2 L3 L4 L5 L6
c
600 400 200
200 400 600 800
Up- regulated
Down- regulated
A B LLC
d
Control
LLC
Z-Score
2 1 0 -1 -2
C1 C2 C3 C4 L1 L2 L3 L4 L5 L6
e
Cumulative Fraction (%)
80 60 40 20
L1 L2 L3 L4 L5 L6
-1 0 1 2 3 4 5 6 7 8
Log2 Fold Change
F
Cumulative Fraction (%)
80 60 40 20
L1 L2 L3 L4 L5 L6
-6 -5 -4 -3 -2 -1 0
Log2 Fold Change
g
Extracellular matrix
PI3K-Akt signaling pathway
Focal adhesion
Cell differentiation
Proteolysis
Angiogenesis
Inflammatory response
Gap junction
PDGF binding
Negative regulation of JAK-STAT
0 5 10 15 20
Fold Enrichment

Figure S3. (**a**) Gene-Ontology analysis of differentially expressed genes (DEG) from control vs. tumor-bearing mice (LLC) for individual clusters (I to IV) identified by the unsupervised hierarchical clustering analysis (shown in Fig. 2B). Each horizontal gray bar represents the gene fold enrichment compared to the total number of genes in each ontology term. (**b**) Hierarchical clustering of the Pearson correlation values identified LLC subgroups represented by colored lines in the dendrogram: control group (light blue; C1-C4), LLC group (pink; L1-L6), subgroup A (red; L2-L4), and subgroup B (black; L5 and L6). (**c**) Bar plot representing the total number of up- and down-regulated genes (red and blue, respectively) in each subgroup of samples identified in LLC (as shown in B). (**d**) Heatmap of 443 Z-score normalized DEG identified in LLC subgroup A (L2-L4) vs. control (C1-C4) analyzed by unsupervised hierarchical clustering. Down- and up-regulated genes with absolute values of fold-change > 1.5 and FDR < 0.05 (Wald test) are shown in red and blue, respectively. Subgroup A- cumulative frequency distribution of the DEG (log2-fold change, x-axis) from LLC (L1-L6) vs. control samples, indicated as a percentage (%, y-axis) for up- and down-regulated genes in (**e**) and (**f**), respectively. (**g**) Gene-Ontology analysis of DEG from control vs. LLC samples from subgroup A. Each horizontal black bar represents the gene fold enrichment compared to the total number of genes in each ontology term.

Figure S4

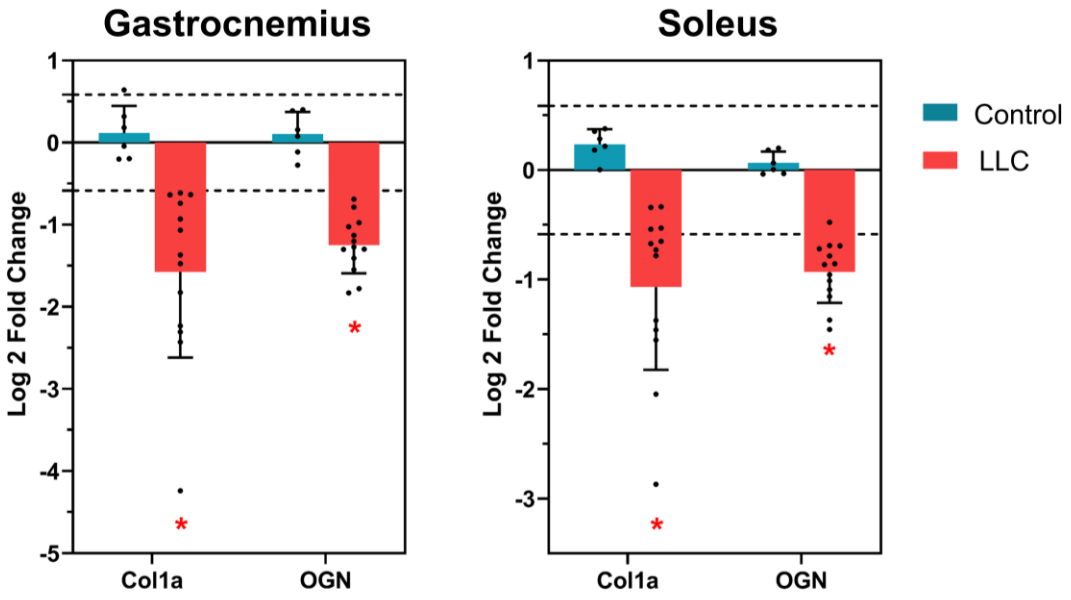

Figure S4. mRNA levels of the genes involved with ECM in different muscle types from LLC and control groups. RT-qPCR data are presented as Log2 fold change (2^-ΔΔCt) relative to Rpl13a. Statistical difference was analyzed by Student's t-test. * $P < 0.05$.
